# Mapping Genomic Regions for Grain Protein Content and Quality Traits in Milled Rice (*Oryza sativa* L.)

**DOI:** 10.3390/plants14060905

**Published:** 2025-03-14

**Authors:** Violina Bharali, Suneetha Yadla, Srinivas Thati, Bhargavi Bitra, Divya Karapati, Neeraja Naga Chirravuri, Jyothi Badri, Raman Meenakshi Sundaram, Aravind Kumar Jukanti

**Affiliations:** 1 Agricultural College, Acharya NG Ranga Agricultural University, Bapatla 522101, India; 2 Regional Agricultural Research Station (RARS), Maruteru 534122, India; 3 ICAR—Indian Institute of Rice Research, Rajendranagar, Hyderabad 500030, India

**Keywords:** grain protein content, QTL mapping, F_2_ population, variability

## Abstract

Grain protein content (GPC) is gaining attention due to increasing consumer demand for nutritious foods. The present study carried out at ICAR-IIRR, Hyderabad, focused on the identification of quantitative trait loci (QTLs) linked with GPC and other quality traits. We utilized a population of 188 F_2_ individuals developed from BPT 5204 (low GPC) X JAK 686 (high GPC) for QTL analysis. QTL analysis yielded four significant QTLs for GPC, three for amylose content, and multiple QTLs for other quality traits. *qPC1.2*, a major QTL in milled rice, was located in the marker interval RM562-RM11307 on chromosome 1 with an LOD value of 4.4. *qPC1.2* explained 15.71% of the phenotypic variance (PVE). Additionally, the Interval Mapping for Epistatic QTLs (IM-EPI) method detected 332 pairs of di-genic epistatic QTLs. Fifteen QTLs exhibited a positive additive effect, indicating that the contributing allele(s) was from JAK 686. Five F_2_ plants, *viz*., F_2_-140, F_2_-12, F_2_-7, F_2_-147, and F_2_-41, exhibited a high GPC of 14.67%, 14.36%, 14.32%, 13.60%, and 13.36%, respectively. Additionally, these plants also exhibited high per-plant grain yield (~17.0–29.0 g) with desirable agronomic traits. The QTLs identified are valuable resources for developing high-grain-protein varieties with high grain yield and desirable quality traits.

## 1. Introduction

Globally, rice is a major staple food crop and is a significant energy source for about half of the world’s population [1]. Rice is a widely consumed cereal, accounting for approximately 21% of human caloric requirements globally [2]. It is mostly produced and consumed in South and Southeast Asia and accounts for up to ~76% of caloric intake in this region. People mostly consume rice in the milled (polished) form, which only consists of endosperm. The processing of hulled/brown rice removes the embryo and bran. The nutritional value of milled rice is largely dependent on the endosperm, which comprises starch (70–80%), proteins (7–10%), and lipids (<1.0%) [3]. The rice endosperm is relatively deficient in many micronutrients or phytonutrients. The total seed protein in rice comprises 60–80% glutelin and 20–30% prolamin [4,5].

Multiple genetic factors with a complex genetic basis control GPC in rice. Environment also has a significant impact on GPC. GPC exhibits wide variation among the different varieties. Among the different *japonica* rice varieties collected from different regions, the GPC ranged from 6.45 to 11.1% (Lou et al. 2023) [6]. Analysis of approximately 4000 rice varieties collected from 57 countries for GPC exhibited a range from 5.3% to 13.6% (Lou et al. 2023). However, the GPC in present-day cultivated varieties ranges from 6.0% to 8.0% only. GPC influences the cooking and eating quality of rice, and affects the physicochemical properties [2]. High GPC causes cooked rice to be hard and loose, resulting in poor eating quality [7]. Consumers usually prefer soft and fluffy cooked rice.

Since GPC is a complex trait influenced by multiple factors, it may not be possible to meet the demands of the growing population using only conventional methods of crop improvement. Breeding approaches like marker-assisted breeding (MAB) are very promising. The application of MAB in crop improvement requires information about the genomic regions governing the trait. The mapping of complex quantitative traits like GPC is relatively difficult compared to simple Mendelian traits. Mapping methods such as QTL mapping have been very effective in identifying the genomic regions governing a particular trait. The effectiveness of QTL mapping depends on precise phenotyping and efficient marker-based genotyping. Rice breeders extensively use thousands of ‘Simple Sequence Repeats (SSRs)’ to perform genotyping across the entire genome [8]. Vast genetic resources are available in rice, including more than 20,000 SSR primers [9]. SSRs are very useful for the construction of linkage maps, mapping of genes, and marker-assisted selection [9].

To date, more than 40 QTLs with small effects on GPC have been identified [10,11,12,13], but very few QTLs have been cloned and functionally validated [14]. *OsASN1*, *OsNAC74*, *OsAUX5*, and *SEMIDWARF1* (*SD1*) are some of the important candidate genes that play a role in rice GPC [15,16,17,18]. Despite the success, two major drawbacks of the QTLs identified so far are as follows: first, the QTLs identified are mostly in brown rice; and second, the parents used in breeding lacked the required variation/difference in GPC. Hence, the identification of stable-GPC QTLs specifically in milled rice, without any undesirable effects on agronomic traits and grain quality, is crucial. This is very essential for the genetic enhancement of the GPC in milled rice and the eating quality of cooked rice.

The objective of the present study is to identify QTLs specifically for GPC in milled rice, and for grain quality traits and yield, in an F_2_ population developed from BPT 5204 X JAK-686. The GPC of BPT 5204 and JAK-686 is ~8.0% and ~12.0%, respectively. BPT 5204 is a very popular variety, with a medium-slender grain type, low GPC, and excellent cooking quality, while JAK-686 is a high-GPC germplasm line, which shows early maturity (110 days). The results of this study will be useful in developing high-yielding, protein-rich varieties with a desirable eating and cooking quality.

## 2. Results

The GPC and cooking quality of rice cultivars are two important traits valued by consumers. Identifying genomic regions governing these traits and their introgression into popular varieties like BPT 5204 through marker-assisted breeding is an effective strategy to develop high-yielding and nutritionally rich cultivars. Therefore, identification and validation of QTLs/genes in different genetic backgrounds and environments is a prerequisite for their large-scale application in a variety of crop improvement programs.

### 2.1. Construction of Genetic Map Using F_2_ Population

**Figure 1:**
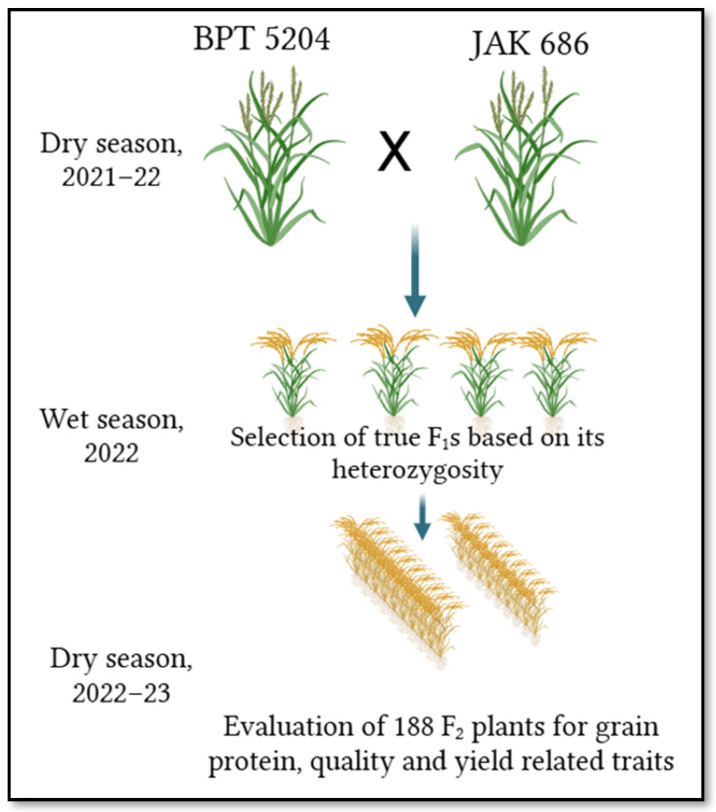
Breeding scheme for development of F_2_ population from BPT 5204 X JAK-686 cross.

**Figure 2:**
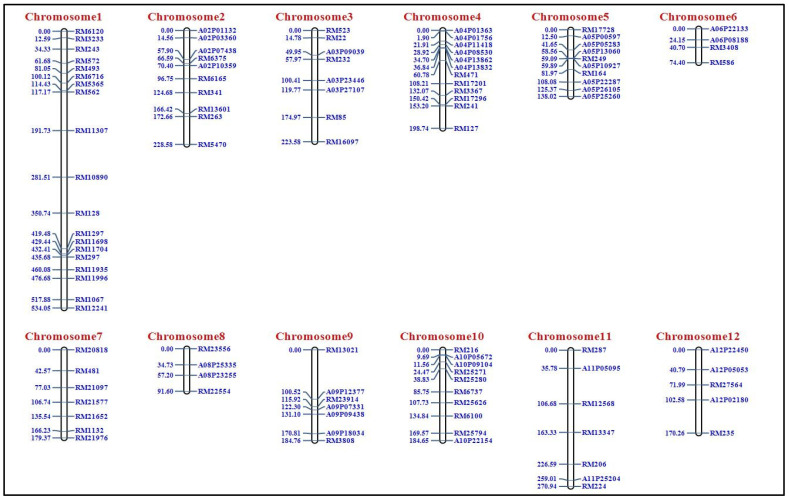
Linkage map generated using IciMapping software V. 4.1 with 103 polymorphic marker.

We developed a population of 188 F_2_ individuals from a cross between “BPT 5204” and “JAK-686” made during the dry season of 2021–2022 (Figure 1). We tested 1220 SSR and 60 InDel markers for polymorphism between parents (BPT 5204 and JAK 686). Supplementary Table S1 lists the differences between the two parents. Among them, 103 markers were polymorphic between BPT 5204 and JAK-686. All 188 F_2_ plants were genotyped using 103 polymorphic markers. The details of the polymorphic markers across the twelve rice chromosomes are listed in Supplementary Table S2. The polymorphic markers were uniformly distributed over all 12 chromosomes, and the highest number of markers was present on chromosome 1 (Table 1). We used the data generated from 103 polymorphic markers on 188 F_2_ plants to construct a genetic linkage map. We used the Chi-square test (*X*^2^) to study the marker segregation distortion. These markers showed a good fit to the expected marker segregation ratio (1:2:1) in a Mendelian fashion. We constructed a genetic map spanning 2478.95 cM with 103 polymorphic marker loci on the 12 linkage groups (Table 1; Figure 2). The highest number of marker loci, nineteen (19), mapped on chromosome 1, at a distance of 534.05 cM. The least featured only four marker loci mapped on chromosomes 6 and 8. The remaining chromosomes had 5–12 marker loci (Table 1).

### 2.2. Identification of QTLs for GPC, Quality Traits, and Yield

**Figure 3:**
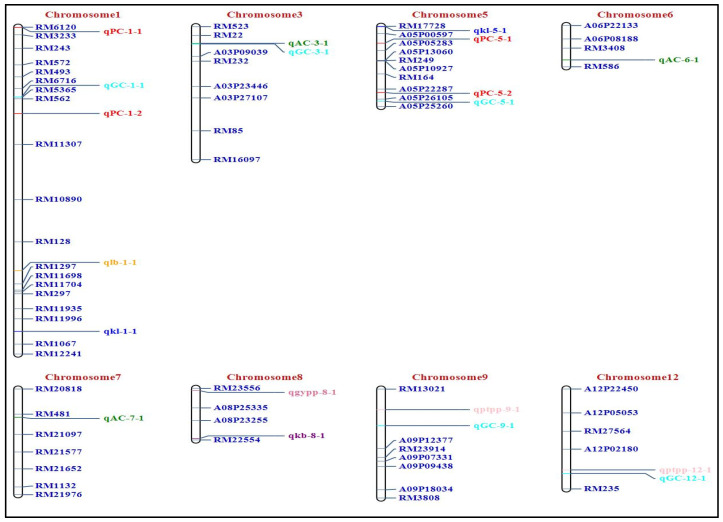
Genetic linkage map indicating position of QTLs for grain protein content, grain quality, yield, and yield traits in F_2_ population.

The focus of the present study was to identify QTLs for GPC, grain quality, and yield using an F_2_ population of BPT 5204 X JAK-686. QTL analysis was performed using the inclusive composite interval mapping additive (ICIM-ADD) method implemented in the ICIM software ICIM [19]. In total, 19 QTLs were identified for GPC, grain quality, yield, and their contributing traits using ICIM-ADD on 12 rice chromosomes (Table 2; Figure 3).

**Figure 4:**
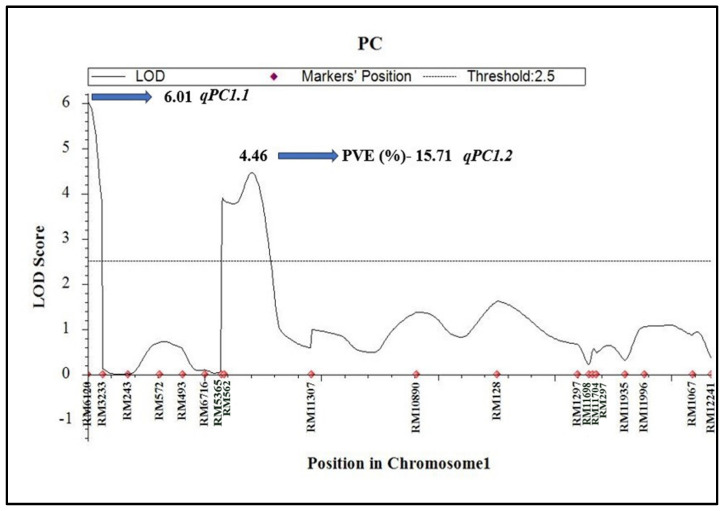
Mapping of major QTL for grain protein content on chromosome 1 using ICIM.

Four QTLs, *qPC1.1* (RM6120-RM3233), *qPC1.2* (RM562-RM11307), *qPC5.1* (A05P00597-A05P05283), and *qPC5.2* (A05P22287-A05P26105), were identified for GP on chromosome 1 and 5, explaining 5.38%, 15.71%, 4.59%, and 3.09% of phenotypic variance (PVE), with LOD scores of 6.01, 4.46, 2.84, and 2.88, respectively (Table 2). Details of previously identified QTLs including their physical location are presented in Table 3. Among the four QTLs for GPC, *qPC1.2*, a major QTL, explained about 15.71% of the PVE (Figure 4). All the four GPC QTLs identified had a positive additive effect, indicating that the trait-enhancing alleles are from JAK-686 (Table 2).

AC and GC, two important quality traits, have a significant impact on the cooking and eating quality of rice. We identified three QTLs for AC (*qAC3.1*, *qAC6.1*, and *qAC7.1*) on chromosome 3, 6, and 7, respectively. *qAC3.1* had the highest LOD score of 6.37, and explained approximately 7.15% of the PVE (Table 2). All the three QTLs identified had positive additive effects, indicating that the contributing allele came from JAK-686. We identified five QTLs (*qGC1.1*, *qGC3.1*, *qGC5.1*, *qGC9.1*, and *qGC12.1*) governing GC. Among the five QTLs, *qGC9.1* and *qGC12.1*, present on chromosomes 9 and 12, respectively, explained about 6.0% of the PVE (Table 2). *qGC9.1* and *qGC12.1* had LOD scores of 3.68 and 3.00, respectively (Table 2).

Additionally, we identified two QTLs for KL: *qkl1.1* and *qkl5.1* on chromosomes 1 and 5, respectively. *qkl1.1* and *qkl5.1*, with LOD scores of 2.86 and 4.77, respectively, explaining 9.63% and 4.24% of the PVE. A single QTL was identified each for KB (*qkb8.1*) and L:B (*qlb1.1*) on chromosomes 8 and 1, respectively. *qkb8.1* and *qlb1.1*, with LOD scores of 3.30 and 2.51, respectively, explained 7.79% and 6.19% of the PVE. We have also identified two QTLs, *qptpp9.1,* and *qptpp12.1*, for PTPP on chromosome 9 and 12, respectively; *qptpp9.1* and *qptpp12.1*, with LOD scores of 4.34 and 3.55, respectively, explained 5.65% and 5.78% of the PVE. Further, *qgypp8.1*, a QTL for GYPP with an LOD value of 2.57, explained 6.07% of the PVE.

### 2.3. Identification of Epistatic QTLs

**Figure 5:**
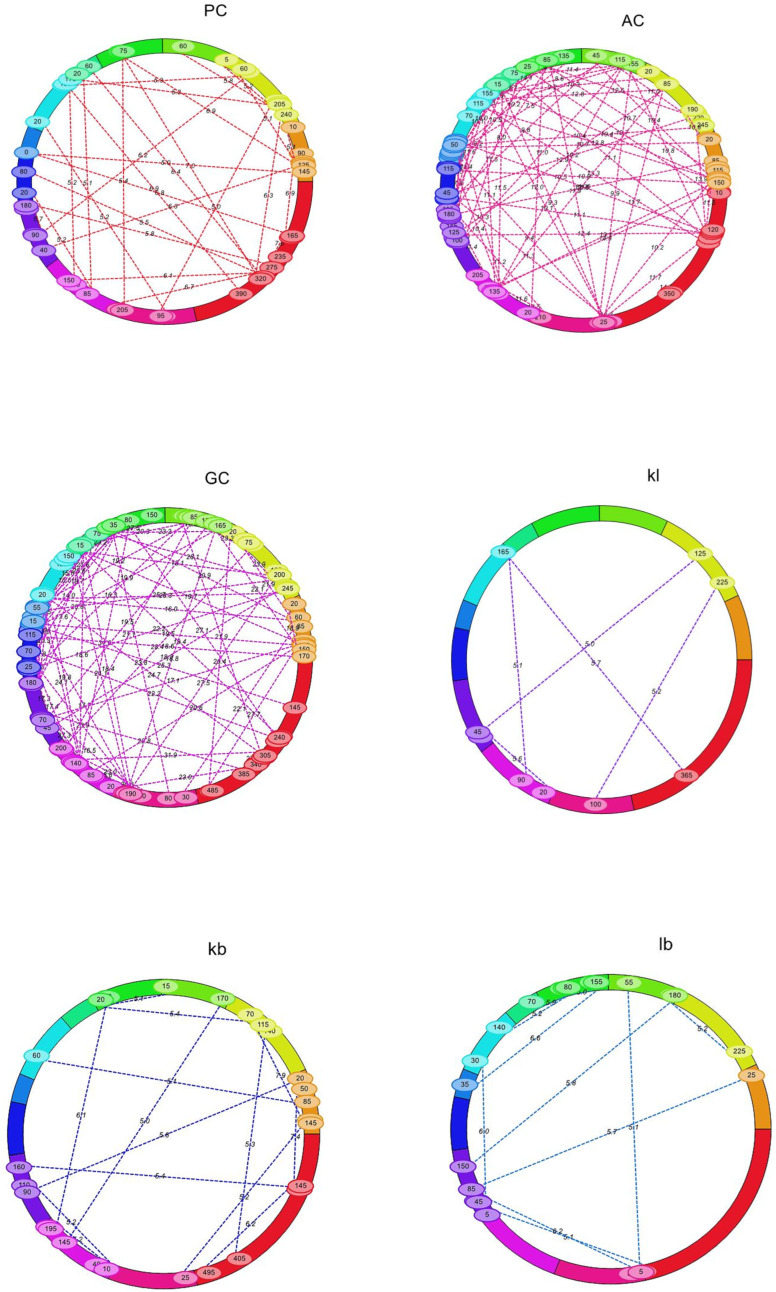

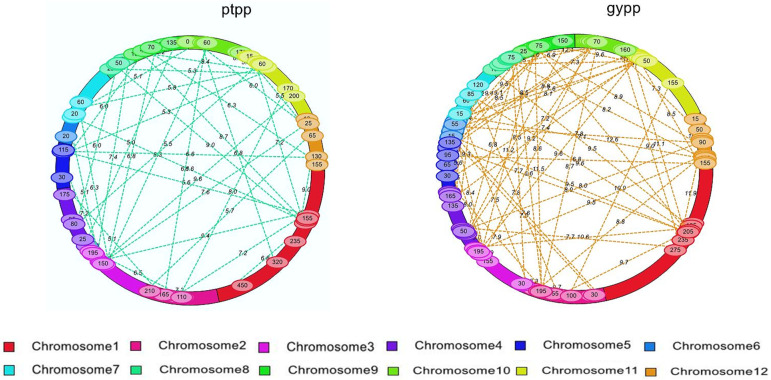
Epistatic QTLs for protein, yield, and quality traits in F_2_ population by IM-EPI (PC—grain protein content; AC—amylose content; GC—gel consistency; kl—kernel length; kb—kernel breadth; lb—L:B; ptpp—productive tillers per plant; gypp—grain yield per plant).

**Figure 6:**
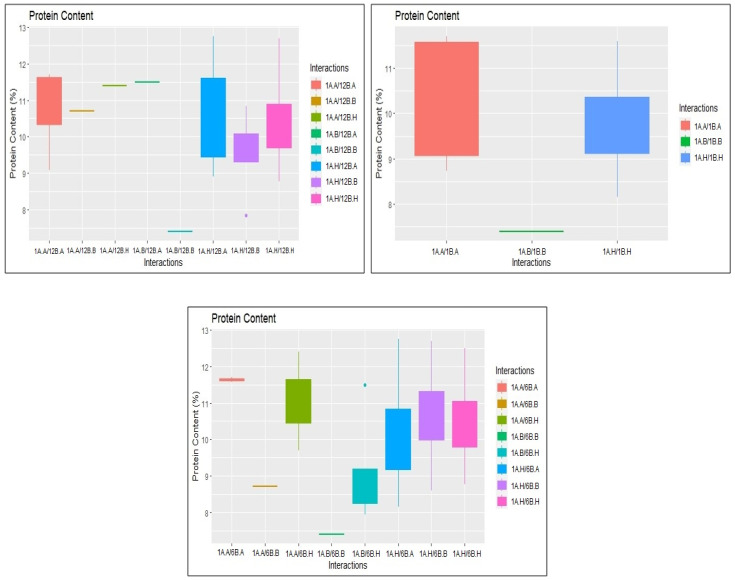
Box plots illustrating the epistatic interactions for protein content on chromosomes 1A (RM562-RM11307) and 12B (RM27564-A12P02180); 1A (RM562-RM11307) and 1B (RM11307-RM10890); 1A (RM562-RM11307) and 6B (A06P22133-A06P08188) A, JAK-686 allele; B, BPT 5204 allele; H, heterozygous.

**Figure 7:**
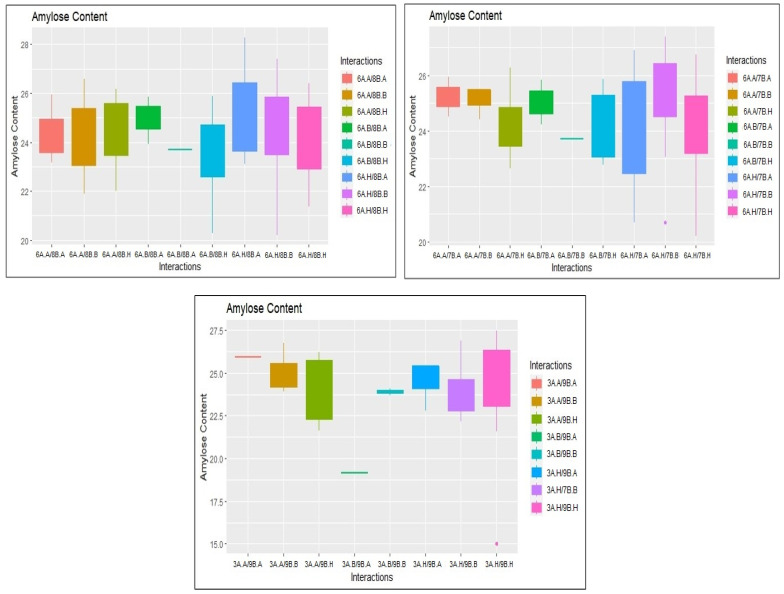
Box plots illustrating the epistatic interactions for amylose content on chromosomes 6A (RM3408-RM586) and 8B (RM23556-A08P25335); 6A (RM3408-RM586) and 7B (RM21652-RM1132); 3A (RM22-A03P09039) and 9B (RM13021-A09P12377).

A total of 331 significant epistatic QTLs were detected for GPC, AC, GC, KL, KB, L:B, PTPP, and GYPP at a threshold LOD score > 5, with PVE ranging from 0.56 to 10.18% (Table S3). At LOD > 5, we found minor interactions between multiple loci across chromosomes (Figure 5). We detected 28 digenic interactions for GPC. Interestingly, three of these interactions occurred between a significant main-effect QTL (RM562-RM11307) in chromosome 1 for GPC and non-significant loci in other chromosomes. These interactions are represented in the boxplots (Figure 6). Both additive effects were from JAK-686 (Table S3). Similarly, we detected 79 epistatic interactions for AC. Three of these interactions occurred between two significant main-effect QTLs (RM22-A03P09039 and RM562-RM11307) on chromosome 3 and 6, respectively, and non-significant loci in other chromosomes (Figure 7). The influence of additive-by-additive effects of epistatic QTLs was relatively lower compared to the individual additive effects of the corresponding QTLs. This could be due to the presence of few individuals with rare loci in the populations. Further, interference from other loci and the limited epistatic variance arising from interactions may also contribute to this effect.

### 2.4. Variability for GPC, Grain Quality Traits, and Yield

**Figure 8:**
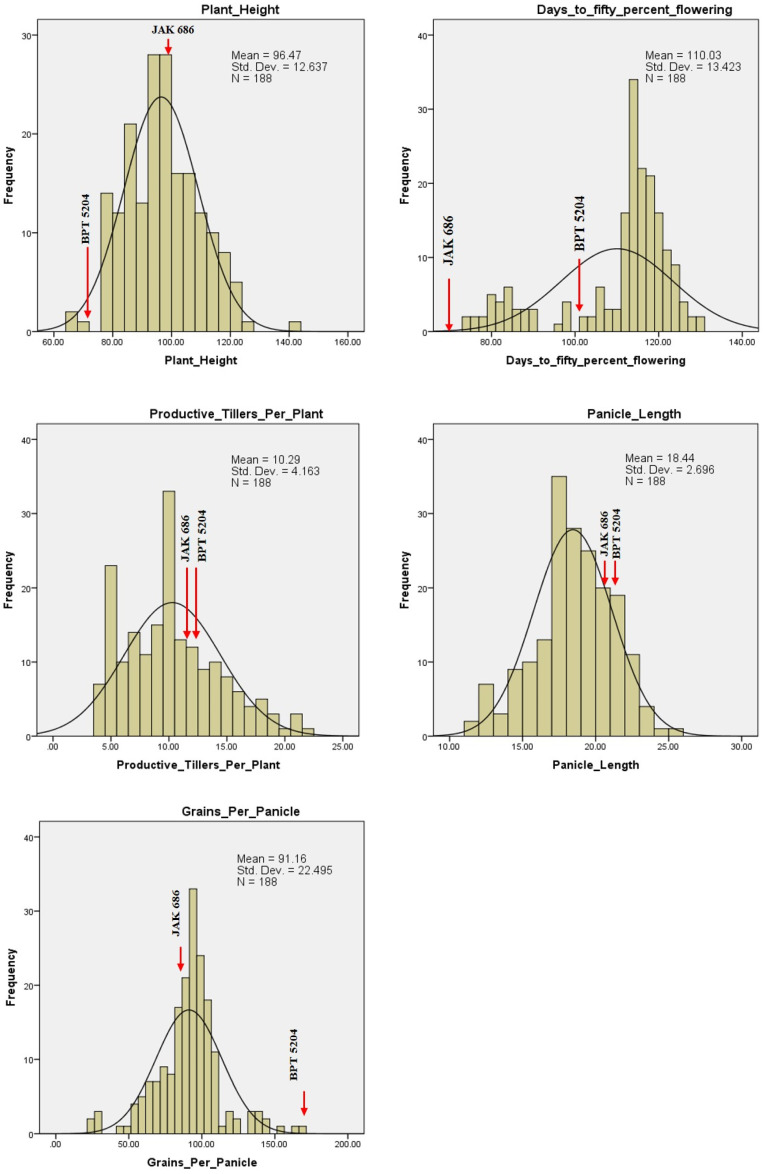
Frequency distribution of F_2_ population for morphological and yield-related traits.

**Figure 9:**
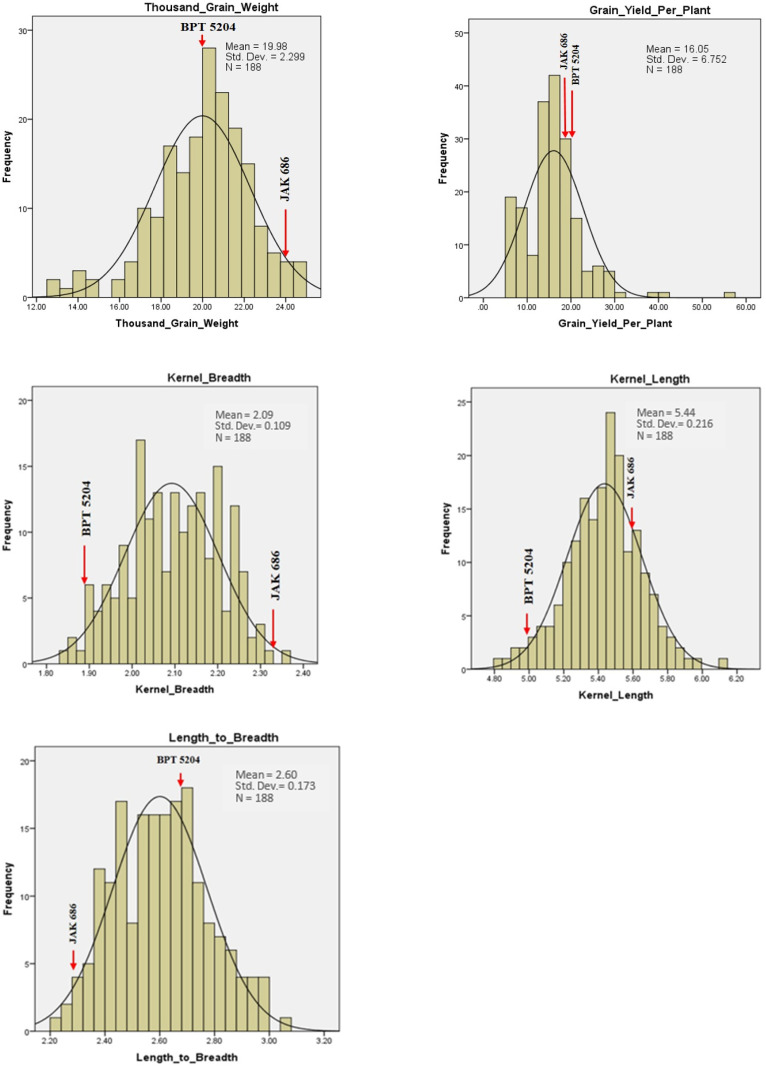
Frequency distribution of F_2_ population for yield and physical quality traits.

**Figure 10:**
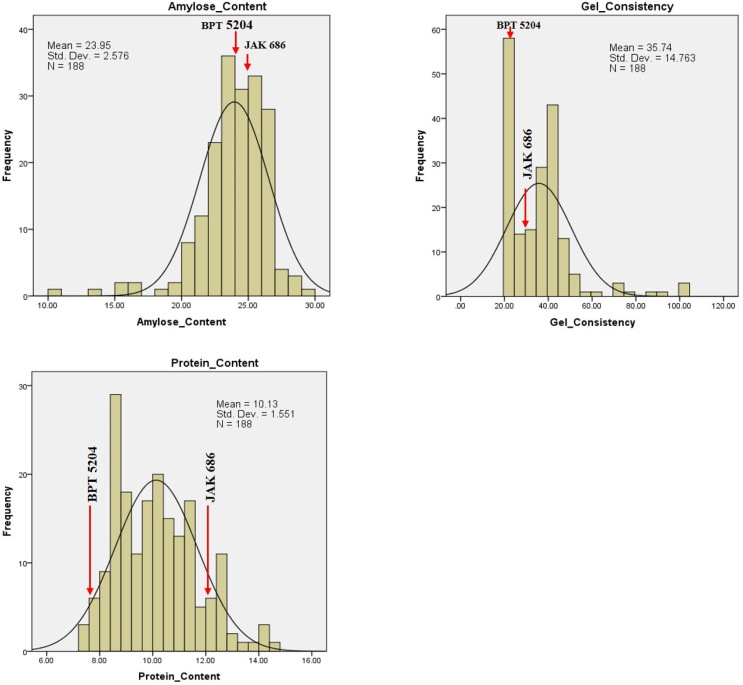
Frequency distribution of F_2_ population for biochemical traits and grain protein content.

Frequency distribution plots for GPC, grain quality, yield, and yield attributing traits showed transgressive segregants for all the traits in one or both directions (Figure 8, Figure 9 and Figure 10). This could be due to the combination of favorable and unfavorable alleles from the parents, ‘BPT 5204’ and ‘JAK-686’. Using phenotypic data from the F_2_ population, a normal distribution and significant variability was observed, suggesting polygenic inheritance of the traits. The GPC ranged between 7.2% (F_2_-186) and 14.7% (F_2_-140), with an average GPC of 10.1% among the F_2_ population. Five F_2_ plants, F_2_-140, F_2_-12, F_2_-7, F_2_-147, and F_2_-41, had high GPC values of 14.67%, 14.36%, 14.32%, 13.60%, and 13.36%, respectively. Additionally, their respective GYPP values were 29.03 g, 19.12 g, 16.54 g, 25.90 g, and 15.87 g (Table 4).

Among the quality traits, GC exhibited the highest variation within the F_2_ population, ranging from 22 mm to 100 mm, with an average GC of 35.7 mm. AC showed a range from 10.14% (in F_2_-127) to 29.01% (in F_2_-66), with an average AC of 24.05%. Among the yield attributing traits, GPP, followed by PH and GYPP, showed the highest variation in the F_2_ population. The values ranged from 24 to 169 for GPP, 65 cm to 143 cm for PH, and 5.12 g to 55.04 g for GYPP.

Correlation studies revealed a negative association of PC with both AC and GC. AC and GC were also negatively correlated (Table 5). AC showed a significant negative correlation with KL and L:B, but a positive correlation with KB. KL had a significant positive correlation with multiple yield-related traits, including GYPP. However, KB showed a significant positive association only with TGW. GPP and TGW exhibited a significant positive correlation with KL, PTPP, and GYPP. GYPP showed a positive and significant association with TGW, GPP, PL, and PTPP.

Among the thirteen traits, the present work revealed high heritability with moderate GAM for TGW (61.4, 14.1), DFF (68.5, 17.3), and AC (75.4, 16.6). We observed high heritability and high GAM for PH (93, 25.3), PTPP (89.2, 71.7), PL (96.4, 28.6), GPP (72.0, 34.3), GYPP (97, 80.8), and GC (90.3, 76.9). The study detected moderate heritability and GAM for GPC (52.4, 16.2). Further, we observed low heritability and GAM for KL (35.1, 2.8), KB (44.9, 4.8), and L:B (33.8, 4.6; Table 6).

## 3. Discussion

The majority of modern-day mega/popular rice cultivars are low in GPC and other essential micronutrients [29,30]. The GPC of milled rice is about 7.0%, whereas brown rice contains 8.0–10.0%. Recent reports have indicated a wide variation for GPC in rice germplasm [30,31]. This existing variability can be exploited for the identification of QTLs, and these QTLs could be utilized through marker-assisted back cross (MABC) breeding to improve high-yielding mega varieties like BPT 5204 (or any other), which have high market acceptability. Both GPC and grain quality can be improved through MABC. The global burden of PEM in 2019 was approximately 148.0 million, ~100.0 million in Asia and 29.0 million in Africa [32]. Interestingly, rice is the major staple diet in these two regions. Hence, any efforts to address the issue of PEM specifically in these regions should include the improvement of GPC along with other nutrients in milled rice. Conventional breeding efforts have had limited success in improving GPC due to its complex nature coupled with the influence of environment. Therefore, exploiting the genetic basis of GPC, coupled with MABC, can aid in developing high-GPC varieties with desirable qualities. Several studies have identified genomic regions affecting GPC, but most of these are for brown rice. Therefore, the present study focused on identifying QTLs for GPC in milled rice, grain quality, and other traits.

BPT 5204 is one of the most popular mega varieties of India. The consumers prefer it due to its excellent eating and cooking quality along with high yield, desirable grain quality, and stable performance across different ecosystems. However, BPT 5204 has a low GPC (7.0–8.0%). In the present study, JAK-686, a high-GPC line (12.5% in milled rice), is the donor parent for GPC. We used the F_2_ population developed from BPT5204 X JAK-686 for identifying genomic regions associated with GPC. Correlation analysis revealed interesting results among the quality traits. AC exhibited a significant negative correlation with GC, indicating that the simultaneous improvement of AC and GC is not possible [33]. However, Roy et al. (2021) [29] reported a positive correlation between AC and GC. Interestingly, PC did not exhibit any significant association with any of the grain quality traits [28]. Suresh et al. (2016) [30] and Roy et al. (2021) [34] reported a positive correlation of KL with yield, and a significant positive correlation with TSW, demonstrating that these traits can be selected and improved simultaneously. GYPP showed a significant positive correlation with yield-related traits like TGW, GPP, PL, and PTPP [34,35,36].

TGW, DFF, and AC exhibited high heritability coupled with moderate GAM. Heritability greater than the genetic advance indicates the influence of environment [37]. We can improve such traits by intermating superior genotypes in the segregating population developed by combination breeding [38]. PH, PTPP, PL, GPP, GYPP, and GC exhibited high heritability and high GAM. Chaudhari et al. (2007) [39] also observed similar results. Since additive gene action largely influences these traits, the potential for their improvement is through direct selection. Accordingly, selection based on these traits is more reliable. GPC exhibited moderate heritability and GAM. This study is consistent with the results of Bruno et al. (2017) [40], suggesting a moderate heritability of protein with higher variation in the population compared to that in the parents due to environmental interaction. Low heritability and GAM were observed for KL, KB, and L:B. These results agree with those reported in other studies [41,42,43]. High heritability coupled with high GAM indicates that most of the heritability is due to additive gene action, making selection potentially effective. Hence, among the thirteen traits studied, selection is effective for PH, PTPP, PL, GPP, GYPP, and GC. Selection for these traits would improve the genotypic value of the selected plants beyond that of their parents [40].

We identified four QTLs (*qPC1.1*, *qPC1.2*, *qPC5.1*, and *qPC5.2*) affecting GPC in milled rice, two each on chromosome 1 and chromosome 5. *qPC1.2*, in the marker interval RM562-RM11307, explained 15.7% of the phenotypic variance. Zhong et al. (2011) [21] reported two QTLs (*qPr1* and *qPr7*) for GPC in milled rice in the marker interval RM493-RM562 and RM445-RM418. The *qPr1* allele from Zhenshan 97B reduced the GPC, while *qPr7* increased the GPC of milled rice. Earlier research has identified numerous QTLs associated with GPC, mostly in brown rice [14,20,44,45]. Several QTLs for GPC in brown rice were identified using different types of populations, including ‘Recombinant Inbred Lines (RILs)’ [27,46] and a ‘Doubled Haploid (DH)’ population [10]. Though these are interesting results, in most of the studies, parents exhibited a narrow range for GPC. In contrast, the parents utilized in the present study showed significant variation for GPC in milled rice, aiding in a more accurate QTL mapping of genomic regions governing GPC. Identifying QTLs in brown rice is useful, but milled rice is more important, as consumers eat brown rice less frequently than milled rice. Further, although researchers have detected numerous QTLs for GPC using different mapping populations, they have characterized only a few. Peng et al. (2014) [25] cloned and validated the *qPC1* locus identified by Wang et al. (2008) [47].

*qPC1* in rice controls GPC by regulating the synthesis and accumulation of different protein fractions [25]. *qPC1* encodes a ‘putative amino acid transporter (*OsAAP6*)’. *OsAAP6* has a positive effect on GPC in rice, indicating that a higher level of expression of *OsAAP6* correlates with higher GPC. *OsAAP6* significantly enhances the root absorption of amino acids and effects their distribution. The palatability of Nangeng 46, a *japonica* cultivar, improves after the introduction of *qPC1* [48]. In addition, *qGPC-10* was cloned and its function was validated [3]. *OsGluA2*, a glutelin type-A2 precursor, is the candidate gene underlying *qGPC-10*. *OsGluA2* functions as a positive regulator of GPC and exerts a pleiotropic effect on the quality of rice grains. *OsGluA2* had a significant effect on GPC and the majority of the protein fractions, with the largest effect on glutelin. The increased or reduced genetic expression of *OsAAP6* and *OsGluA2* would be the primary targets for high/low GPC breeding [14]. Consumers generally consider high GPC as an indicator of superior nutritional value. However, researchers have found a negative correlation between high GPC and eating quality [14]. High GPC in rice results in a compact endosperm, which negatively affects its palatability.

Amylose content (AC) and gel consistency (GC) are two important traits that affect both the cooking and eating quality of rice. Based on AC, rice is classified into glutinous (sticky) and non-glutinous (non-sticky) types. Consumer demands differ widely based on the stickiness of the rice. Numerous studies have underscored the importance of AC and GC. Several QTLs influencing grain quality traits were identified in both *indica* and *japonica* rice. We have identified three QTLs (*qAC 3.1*, *qAC 6.1,* and *qAC 7.1*) for AC. Cheng et al. (2014) [49] detected three QTLs on chromosomes 3, 5, and 6. Earlier studies reported multiple QTLs in chromosome 1 for AC [50,51,52,53]. The *Wx* locus is the major determinant of AC. The *Wx* locus encodes the ‘Waxy’ gene, the major candidate gene responsible for the amylose content in rice [54]. Allelic variations in the *Wx* gene play a crucial role in determining amylose content in rice grains and act as key regulatory factors influencing eating and cooking quality. The *Wx^a^* and *Wx^lv^* alleles are associated with AC levels exceeding 25%, whereas *Wx^in^* exhibits AC levels ranging from 18% to 22%. The *Wx^b^* allele corresponds to AC levels between 15% and 18%. Additionally, alleles such as *Wx^mw^*, *Wx^la^*, *Wx^mq^*, *Wx^mp^*, *Wx^op^*, and *Wx^hp^* demonstrate AC levels between 10% and 15%. Among all variants, the *wx* allele exhibits the lowest AC level, approximately 2% [55].

We have identified five QTLs (*qGC1.1*, *qGC3.1*, *qGC5.1*, *qGC9.1,* and *qGC12.1*) for GC. Among the five QTLs, *qGC1.1* and *qGC3.1* have a negative additive effect, indicating that the contributing allele(s) came from BPT 5204. For the other QTLs, the contributing allele came from JAK 686, the high-GPC parent. Multiple QTLs for GC have been reported across the rice genome using different mapping populations [56,57,58]. Researchers have identified the *Wx* locus on chromosome 6 as the primary determinant of GC, explaining 38.9% of the variation. Further, QTL studies for AC and GC have revealed that the *Wx* and *Alk* loci, located on short arm of chromosome 6, control these traits along with gelatinization temperature [47]. Two QTLs for KL, *qkl1.1* and *qkl5.1*, were identified on chromosomes 1 and 5. Similarly, Singh et al. (2012) [59] identified a KL QTL on chromosome 1 linked to the marker RM 431.

Rabiei et al. (2004) [60] identified five QTLs on chromosomes 2, 3, 5, 7, and 8, with each explaining more than 10% of the PVE. For KB, one QTL, *qkb8.1*, was identified on chromosome 8. Rabiei et al. (2004) [60] detected a QTL, *qGW8*, controlling KB on chromosome 8. Fine mapping of this QTL identified *OsSPL16*, squamosa promoter-binding protein-like 16, and a member of the SBP domain-containing transcription factors [61]. *OsSPL16* encodes a protein that acts as a positive regulator of cell proliferation. A higher level of expression of *OsSPL16* promotes cell division and has a positive impact on grain width. On the contrary, a loss-of-function mutation of *OsSPL16* in basmati improves grain quality by producing more slender grains. Song et al. (2007) [62] successfully cloned the QTL *GW2* (Grain Width on Chromosome 2), which encodes a RING-type E3 ubiquitin ligase that regulates rice grain width. *GW2* functions as a negative regulator of cell division by directing its substrate for proteasomal degradation. The loss of function in *gw2* leads to an increase in cell numbers within the spikelet hull, thereby contributing to greater grain width, weight, and yield. The major QTL, *qSW5/GW5* (Seed Width/Grain Width on Chromosome 5), plays a crucial role in determining grain width and has been mapped to a 21–22 kb genomic region [63,64]. *GW5* functions within the brassinosteroid signaling pathway, modulating grain development by inhibiting the kinase activity of *GSK2* on *OsBZR1* and *DLT*, thereby regulating grain width [65].

*qlb1.1*, a QTL for kernel length-to-breadth (L:B), was identified on chromosome 1. *qlb 1.1* exhibited positive additive effects, indicating that the contributing allele came from JAK-686. Rabiei et al. (2004) [60] identified QTLs controlling grain shape (based on L:B) on chromosomes 2, 3, 5, 7, and 8, while Chen et al. (2021) [66] identified *qGS7*. *qGS7* contained the gene *GL7*/*GW7*, characterized by Wang et al. (2015) [67]. The upregulation of *GW7* expression increased the production of slender grains, thereby enhancing the grain’s appearance and quality. Zhao et al. (2018) [68] identified a QTL, *GS9* (Grain Shape Gene on Chromosome 9), that negatively influences the length-to-width ratio of rice grains. *GS9* regulates grain morphology by modulating horizontal cell division and vertical cell elongation, thereby affecting overall grain shape. *qlb1.1*, identified in this study, appears to be a novel QTL, as no loci for L:B have been reported earlier on chromosome 1. Zhao et al. (2022) [2] have identified a significant QTL for PTPP on Chromosome 5, while the present work identified QTLs for PTPP on chromosome 9 and 12. Interestingly, a significant QTL for grain yield was identified on chromosome 8 [61], supporting the present findings. Another study identified *cytochrome b561* as the gene underlying *qGY8.1*, a QTL for yield [69]. *Cytochrome b561* belongs to a protein family that plays an important role in plant growth and development, particularly under drought conditions.

We identified QTLs for GPC, quality traits, and yield, which can be used for fine mapping and the identification of specific candidate genes for these traits. This information can used to develop functional markers for trait improvement through marker-assisted backcross breeding (MABC). Additionally, the present findings suggest that further analysis is needed to fully understand the genetic basis of the significant variation observed among the studied traits.

## 4. Materials and Methods

### 4.1. Plant Material

BPT 5204 (female) and JAK-686 (male) are the two diverse parents (for grain protein content) used in the development of the F_2_ population. BPT 5204 is a high-yielding popular variety with medium-slender grain, an excellent cooking and eating quality, but with low grain protein content (7.0–8.0% in milled rice). JAK-686 is an early maturing and high-grain-protein-content (~12.5%) germplasm line. We developed a population of 188 F_2_ individuals from a cross between “BPT 5204” and “JAK-686” made during the dry season of 2021–2022. True F_1_s were selfed in the wet season of 2022 to generate 188 F_2_ individual plants. During the dry season of 2022–2023, the phenotyping and genotyping of 188 F_2_ plants for protein content, quality trait yield, and their component traits was completed. The field experiments were conducted at the research farm of ICAR—Indian Institute of Rice Research, Hyderabad.

### 4.2. Grain Protein Content, Grain Quality, Yield, and Yield Attributing Traits

The transplanting of F_2_ plants occurred at 25 days after sowing with an interrow spacing of 20 cm and an interplant spacing of 15cm. Following the recommended agronomic practices and pest control measures ensured a healthy crop. The F_2_ population was evaluated for seven yield and yield attributing traits, *viz*., plant height (PH; cm), productive tillers per plant (PTPP), panicle length (PL; cm), grains per panicle (GPP), days to 50% flowering (DFF), 1000-grain weight (TW; g), and grain yield per plant (GYPP; g). Screening of F_2_ mapping population was also carried out for GPC (%) and quality traits, *viz*., kernel length (KL; mm), kernel breadth (KB; mm), length:breadth ratio (L:B), gel consistency (GC; mm), and amylose content (AC; %). We estimated the nitrogen content of milled rice through the micro-Kjeldahl distillation method [70]. The nitrogen value thus obtained was converted to protein content by multiplying it by a correction factor of 6.25 [71]. Genetic variability parameters like the Genotypic Coefficient of Variation (GCV) and Phenotypic Coefficient of Variation (PCV) were calculated as per Burton and Dewane (1953) [72], along with heritability and genetic advance as a percentage (GAM) [73,74]. The correlations were determined using R software version 1.4.1717 while the frequency distribution plots were derived using IBM SPSS Statistics 20.

### 4.3. QTL Analysis

We extracted DNA from leaf samples using the CTAB method and assessed its quality and quantity using a UV spectrophotometer at both 260 nm/280 nm and 260 nm/230 nm. Genomic DNA was isolated from 188 fresh leaf samples using the CTAB method, and the F_2_ population was genotyped. We used a working DNA concentration of 40 ng/µL for genotyping. A set of 1280 markers, comprising 1220 SSRs and 60 InDels, evenly distributed across the rice genome were used to study parental polymorphism. The SSR and InDel markers which have been previously reported in the universal core genetic map for rice [75], the genome-wide InDel marker set for allele discrimination between rice (*Oryza sativa*), and the other seven AA-genome *Oryza* species [76], respectively, were used for the polymorphism study between parents. Some SSR markers for the rice genome were retrieved from the Gramene Marker Database (https://gramene.org/archive (accessed on 27 December 2022)). Markers were selected from all 12 chromosomes of rice (*Oryza sativa* L.) to ensure comprehensive genome-wide coverage.

Among the 1280 markers, 103 were polymorphic. We screened the F_2_ population using these 103 polymorphic markers. PCR was carried out in a Bio-Rad T100^TM^ Thermal Cycler (Bio-Rad, Hercules, CA, USA) with a reaction volume of 10 µL, containing 15 ng of genomic DNA, primers at concentrations ranging from 5 to 10 pM, and Emerald Amp GT PCR Master Mix (Takara Bio India, New Delhi, India). The details of PCR cycles are as follows: initial denaturation at 94 °C for 5 min, followed by 35 cycles of denaturation at 94 °C for 30 s, annealing at 55 °C for 30 s, extension at 72 °C for 1 min, and a final extension step at 72 °C for 10 min. Amplified products were separated using 3% agarose gel electrophoresis in 1 × TAE buffer at 120 V for 2 h. Ethidium bromide staining was used to visualize bands and a gel documentation system was employed to document the results.

SSRs and InDels were scored as co-dominant markers, with “A” representing the presence of the JAK-686 allele, “B” representing the presence of the BPT 5204 allele, and “AB” indicating heterozygous bands. A Chi-square test was conducted to assess the segregation ratio in the F_2_ mapping population. We then constructed a linkage map using genotypic data from 188 F_2_ plants and 103 polymorphic markers spanning all the rice chromosomes. We developed the genetic linkage map using ICIM and utilized Kosambi’s mapping function to convert recombination frequencies into centiMorgan (cM). QTL mapping was carried out through inclusive composite interval mapping of additive QTLs (ICIM-ADD) and inclusive composite interval mapping of epistatic QTLs (ICIM-EPI), implemented in QTL IciMapping v4.1. We considered a QTL significant if it exceeded the logarithm of odds (LOD) threshold of 2.5, determined through a 1000-permutation test. Stepwise regression analysis was utilized to estimate the percentage of phenotypic variance explained (PVE or R^2^) by individual QTLs and to determine their additive effects at the LOD peak positions.

## 5. Conclusions

Improving the grain protein content in rice is essential for addressing protein energy malnutrition, particularly in developing nations. In the present study, we have successfully identified four QTLs for GPC and three for amylose content in milled rice. *qPC1.2*, the major QTL, was located on chromosome 1, explaining about 15.7% of the phenotypic variation. Further characterization of the identified GPC QTLs will aid in identification of the candidate gene(s). Subsequently, this information on genomic regions will be useful in the introduction of high-GPC traits into elite high-yielding backgrounds. Additionally, the F_2–3_ progeny with 13–14% protein content identified in the present study can be stabilized through generation advancement (F_5_ or F_6_). The stable material will have a high GPC, coupled with a high grain yield and desirable grain and cooking quality. This cultivar can fetch additional income for farmers as it is of premium grain and nutritional quality. Overall, the present study successfully identified valuable genomic resources for GPC and other quality traits in milled rice that will be useful for researchers, farmers, and consumers.

## Figures and Tables

**Table 1:** Details of markers used and development of linkage map.

S. No.	Chromosome	Total Markers	Polymorphic Markers	Polymorphism (%)	Length (cM)
1	1	185	19	10.27	534.05
2	2	94	10	10.63	228.58
3	3	168	8	4.76	223.58
4	4	119	12	10.08	198.74
5	5	92	10	10.86	138.02
6	6	112	4	3.57	74.40
7	7	96	7	7.29	179.37
8	8	89	4	4.49	91.60
9	9	95	7	7.36	184.76
10	10	61	10	16.39	184.65
11	11	68	7	10.29	270.94
12	12	101	5	4.95	170.26
	Total	1280	103	8.04	2478.95

**Table 2:** Additive QTLs detected for protein content, grain quality, and yield in F_2_ population.

S. No.	Trait	CHR	QTL	Position	Marker Interval	LOD	PVE(%)	Add	Parental Allele
1	PC	1	*qPC1.1*	0	RM6120-RM3233	6.01	5.38	0.67	J
2	PC	1	*qPC1.2*	141	RM562-RM11307	4.46	15.71	1.28	J
3	PC	5	*qPC5.1*	29	A05P00597-A05P05283	2.84	4.59	0.20	J
4	PC	5	*qPC5.2*	114	A05P22287-A05P26105	2.88	3.09	0.57	J
5	AC	3	*qAC3.1*	28	RM22-A03P09039	6.37	7.15	4.10	J
6	AC	6	*qAC6.1*	62	RM3408-RM586	3.25	2.71	1.36	J
7	AC	7	*qAC7.1*	48	RM481-RM21097	2.91	1.52	0.66	J
8	GC	1	*qGC1.1*	114	RM6716-RM5365	3.57	1.22	−6.00	B
9	GC	3	*qGC3.1*	29	RM22-A03P09039	5.56	6.23	−21.56	B
10	GC	5	*qGC5.1*	129	A05P26105-A05P25260	3.35	1.27	1.57	J
11	GC	9	*qGC9.1*	62	RM13021-A09P12377	3.68	5.90	12.64	J
12	GC	12	*qGC12.1*	144	A12P02180-RM235	3.00	5.89	12.37	J
13	KL	1	*qkl1.1*	497	RM11996-RM1067	2.86	9.63	−0.0005	B
14	KL	5	*qkl5.1*	0	RM17728-A05P00597	4.77	4.24	0.07	J
15	KB	8	*qkb8.1*	89	A08P23255-RM22554	3.30	7.79	0.04	J
16	L:B	1	*qlb1.1*	398	RM128-RM1297	2.51	6.19	0.002	J
17	PTPP	9	*qptpp9.1*	35	RM13021-A09P12377	4.34	5.65	3.55	J
18	PTPP	12	*qptpp12.1*	138	A12P02180-RM235	3.55	5.78	3.63	J
19	GYPP	8	*qgypp8.1*	4	RM23556-A08P25335	2.57	6.07	−2.30	B

PC = protein content, AC = amylose content, GC = gel consistency, KL = kernel length, KB = kernel breadth, PTPP = productive panicles per plant, GYPP = grain yield per plant.

**Table 3:** QTLs identified for grain protein content.

Trait	Chromosome	Identified QTLs		Previously Known QTLs		References
		Name	Physical Position (Mbp)	Name	Physical Position (Mbp)	
Protein content	1	*qPC1.1*	4.31–5.05	*qPC1.1*	4.63–4.70	[20]
	1	*qPC1.2*	14.61–23.92	*qPr1*	12.20–14.63	[21]
	1			*qGPC1.1*	0.6–1.1	[22]
	1			*qPC1.1*	8.07	[23]
	1			*qRPC-1*	11.07	[11]
	1			*qPC-1*	25.02–26.19	[24]
	1			*Pro-1*	32.09–34.02	[10]
	1			*qPC1*	37.88–40.16	[25]
	1			*qPC1.2*	39.16–39.23	[20]
	1			*qPC1*	40.13–41.16	[26]
	5	*qPC5.1*	0.59–5.28	*qPC5*	1.94	[26]
	5	*qPC5.2*	22.28–26.10	*qPC-5*	23.48–24.26	[27]
	5			*qPC5.1*	0.53	[28]
	5			*qGPC5*	7.8	[2]

**Table 4:** Characterization of five (5) promising F_2_ lines for GPC, grain quality, yield, and yield attributing traits.

Genotypes	GPC (%)	PH (cm)	PTPP	PL (cm)	GPP	TGW (g)	GYPP (g)	DFF	AC (%)	GC (mm)	KL (mm)	KB (mm)	L:B
F_2_-140	14.67	93	18	21	126	24.5	29.03	114	25.87	22	5.65	2.18	2.59
F_2_-12	14.36	91	10	16.83	90	21.3	19.12	119	23.46	49	5.74	1.98	2.89
F_2_-7	14.32	85	21	20.27	82	18.4	16.54	119	23.17	24	5.5	2.02	2.72
F_2_-147	13.60	104	10	19.4	155	23.6	25.90	115	23.61	48	5.47	2.28	2.39
F_2_-41	13.36	95	12	19.43	103	23.6	15.87	116	21.88	52	6.1	2.11	2.89

GPC = grain protein content; AC = amylose content; GC = gel consistency; KL = kernel length; KB = kernel breadth; L:B = length-to-breadth ratio; PH = plant height; PTPP = productive tillers per plant; PL = panicle length; GPP = grains per panicle; TGW = 1000-grain weight; GYPP = grain yield per plant; DFF = days to 50% flowering.

**Table 5:** Correlation studies in F_2_ population of BPT 5204 X JAK-686 cross.

Traits	PC	AC	GC	KL	KB	L:B	PH	PTPP	PL	GPP	TGW	GYPP	DFF
PC	1	−0.060	−0.054	0.104	−0.019	0.082	−0.049	0.019	0.087	0.078	0.052	0.025	0.064
AC	−0.060	1	−0.245 **	−0.135 *	0.143 *	−0.187 **	0.021	−0.102	0.029	−0.023	0.075	−0.044	−0.043
GC	−0.054	−0.245 **	1	0.024	0.090	−0.057	−0.034	0.000	0.089	0.074	−0.036	0.135 *	0.088
KL	0.104	−0.135 *	0.024	1	0.014	0.585 **	−0.068	0.305 **	0.208 **	0.187 **	0.258 **	0.263 **	0.049
KB	−0.019	0.143 *	0.090	0.014	1	−0.801 **	0.133 *	−0.056	−0.012	−0.043	0.389 **	0.005	−0.047
L:B	0.082	−0.187 **	−0.057	0.585 **	−0.801 **	1	−0.145 *	0.238 **	0.146 *	0.154 *	−0.163 *	0.160 *	0.062
PH	−0.049	0.021	−0.034	−0.068	0.133 *	−0.145 *	1	−0.158 *	−0.047	−0.149 *	0.003	−0.130 *	−0.507 **
PTPP	0.019	−0.102	0.000	0.305 **	−0.056	0.238 **	−0.158 *	1	0.503 **	0.456 **	0.166 **	0.651 **	0.100
PL	0.087	0.029	0.089	0.208 **	−0.012	0.146 *	−0.047	0.503 **	1	0.390 **	0.038	0.487 **	0.078
GPP	0.078	−0.023	0.074	0.187 **	−0.043	0.154 *	−0.149 *	0.456 **	0.390 **	1	0.137 *	0.836 **	0.159 *
TGW	0.052	0.075	−0.036	0.258 **	0.389 **	−0.163 *	0.003	0.166 **	0.038	0.137 *	1	0.125 *	0.044
GYPP	0.025	−0.044	0.135 *	0.263 **	0.005	0.160 *	−0.130 *	0.651 **	0.487 **	0.836 **	0.125 *	1	0.109
DFF	0.064	−0.043	0.088	0.049	−0.047	0.062	−0.507 **	0.100	0.078	0.159 *	0.044	0.109	1

PC = protein content; AC = amylose content; GC = gel consistency; KL = kernel length; KB = kernel breadth; L:B = length-to-breadth ratio; PH = plant height; PTPP = productive tillers per plant; PL = panicle length; GPP = grains per panicle; TGW = 1000-grain weight; GYPP = grain yield per plant; DFF = days to 50% flowering.* Significant at 5% level; ** significant at 1% level.

**Table 6:** Genetic variability in F_2_ population of BPT 5204 X JAK-686 cross.

Characters	Vg	Vp	Ve	PCV	GCV	h^2^ BS	GA	GAM
Plant height (cm)	149.2	160.4	11.2	13.2	12.7	93.0	24.2	25.3
Productive tillers per plant (No.)	15.1	16.9	1.8	39.0	36.8	89.2	7.5	71.7
Panicle length (cm)	7.0	7.2	0.2	14.4	14.1	96.4	5.3	28.6
Grains per panicle (No.)	357.4	496.2	138.7	23.1	19.6	72.0	33.0	34.3
1000-grain weight (g)	3.1	5.0	1.9	11.1	8.7	61.4	2.8	14.1
Grain yield per plant (g)	42.7	44.0	1.2	40.4	39.8	97.0	13.2	80.8
Kernel length (mm)	0.016	0.045	0.029	3.9	2.3	35.1	0.15	2.8
Kernel breadth (mm)	0.005	0.012	0.017	5.2	4.8	44.9	0.10	4.8
Length-to-breadth ratio	0.01	0.029	0.019	6.6	3.8	33.8	0.12	4.6
Days to 50% flowering	125.1	182.6	57.4	12.3	10.2	68.5	19.1	17.3
Gel consistency (mm)	191.2	211.6	20.4	41.3	39.2	90.3	27.0	76.9
Protein content (%)	1.2	2.3	1.1	15.1	10.9	52.4	1.6	16.2
Amylose content (%)	4.9	6.6	1.6	10.6	9.2	75.4	4.0	16.6

Vg = genotypic variance, Vp = phenotypic variance, Ve = environmental variance, PCV = phenotypic coefficient of variation, GCV = genotypic coefficient of variation, h^2^ BS = broad-sense heritability, GA = genetic advance, GAM = genetic advance as percent of mean.

## Data Availability

Data are available from the authors upon reasonable request.

## Supplementary Materials

The following supporting information can be downloaded at: https://www.mdpi.com/article/10.3390/plants14060905/s1, Table S1: Details of both parents used for developing F_2_ population; Table S2: Details of 103 polymorphic markers; Table S3: Details of Di-Genic epistatic QTLs detected for GPC, grain quality and yield traits in F_2_ population.
