# Supplementary material for: Mapping Genomic Regions for Grain Protein Content and Quality Traits in Milled Rice (Oryza sativa L.)
Source: Plants (Basel). 2025 Mar 14;14(6):905. doi: 10.3390/plants14060905 (PMC11944466; doi:10.3390/plants14060905)
Supplement: Supplementary file 1 [file plants-14-00905-s001.zip › plants-3470218-supplementary.pdf]

Table S1. Details of both parents used for developing F_2 population

S. No.	Characters	BPT 5204	JAK-686
1	Maturity Duration (Days)	140-145	100-110
2	Plant height (cm)	73-76	97-103
3	Grain type	Medium slender	Short bold
4	Grain yield per plant (g)	20	18
5	Length to Breadth ratio	2.7	2.2
6	Test weight (g)	14.7	23.0
7	Protein content (%)	7.1	12.5
8	Amylose content (%)	23.0	26.0

Table S2. Details of 103 polymorphic markers

S. No	Marker	Chromosome	Forward (5′ to 3′)	Reverse (5′ to 3′)	Product size	Tm (°C)	Ta (°C)
1	RM6120	1	CAGACGCTTTAATAGCTGGTATGTGC	CCCTACGACGCCTACAGCTACG	445	65.5	61.2
2	RM3233	1	GAAATTCGAAATGGAGGGAGAGC	GGTGAGTAAACAGTGGTGGTGAGC	132	64.4	61.2
3	RM243	1	CAGACTGCAGTTGCACGATACTACG	GAAAGCTGCAACGATGTTGTCC	112	61.6	61.6
4	RM572	1	CGCGGTTAATGTCATCTGATTGG	CCATACTTCGAGATCCAAGACTGACC	168	63.4	60.9
5	RM493	1	GTACGTAAACGCGGAAGGTGACG	CGACGTACGAGATGCCGATCC	178	64.7	64.7
6	RM5365	1	CAAAGTTCGGATCATCTGTTCG	ACATATCAGGGAAAGCGTCAGG	153	61.2	59.1
7	RM562	1	GGAAAGGAAGAATCAGACACAGAGC	GTACCGTTCCTTTCGTCACTTCC	126	63.1	62.4
8	RM10890	1	GCTTCGGCTCTTCATTCACTGG	GCGATTATAGGAGCGCTATGTGG	241	61.2	61.2
9	RM6716	1	CCTATGCGATCTCCTATTTGG	CGAGTCAGACACGTACTACTGC	160	61.2	57
10	RM11307	1	AAAGCTCTGCAATCTTCTCTCC	GAATACGACATCAGAACAGTGC	148	58.1	58.1
11	RM1297	1	AACGCAGTGCAAGTTGTATAGTGC	TCAGTACGTCCGGTCTGAAAGC	179	63.7	61.2
12	RM128	1	TGATTTCTTGGAAGCGAAGAGTGAGG	CCTCCTTGTGCTCAGCCATGC	95	65.2	63.5
13	RM11698	1	ATCAGCATCCCAAAGCTAGAACC	AACCGTATATTGAGGGAGCAAGC	123	61.1	61.1

S. No	Marker	Chromosome	Forward (5′ to 3′)	Reverse (5′ to 3′)	Product size	Tm (°C)	Ta (°C)
14	RM11704	1	ACAGGGCTATGCAGACACAGTGC	AGCAAGCGAAGGGAAGTGACC	241	64.5	64.5
15	RM297	1	ACAGGGCTATGCAGACACAGTGC	AGCAAGCGAAGGGAAGTGACC	241	64.5	61.4
16	RM11935	1	AGCTGACGCCTGGACTATGAAGC	CACAGCCTAAGTCTCTGTCAACATCC	163	63.4	63.4
17	RM11996	1	CCGTCGTCAATATTTCTACGACACC	GGCTTAAATTGCCACTGTTCAGG	147	61.2	61.2
18	RM12241	1	GGATACTCTATGTTCCTCTGCTGTGC	GTCTCCGCCGTCTCGTAAACC	148	65.3	62.7
19	RM1067	1	CGATCCAGAGAGAATGTCTAG3	TAATACGCAAGGCAGAAGGG3	160	56.5	56.4
20	A02P01132	2	GAGACGCAACCACGACATCA	CATAGGCATATGACTGGAGG	217	53.4	53.4
21	A02P03360	2	ACATCATCCAAATGTTCACT	GGGTGTTTCACTGATTTGC	202	54.5	51.2
22	A02P07438	2	TGCTCCATGTGGAGAAGGC	GTGCAGCTCTGGTTGCAGT	135	59.2	59.2
23	RM6375	2	CGAATGGAACGACAAGAGATGC	ATAGATCGACAACGTAGATCCACAGG	160	61.9	61.1
24	A02P10359	2	AGTTCGTCTACGGCTCCGT	GTCGATCTCGTCGTGCTTG	257	58.2	58.2
25	RM6165	2	TCCTTCTCCTTGAACAGCGACAGC	AGAGGCGAGAGGCATGGAGTGC	171	67.7	65.6
26	RM341	2	CAAGAAACCTCAATCCGAGC	CTCCTCCCGATCCCAATC	150	58	56.3
27	RM13601	2	ACGACCTGGGTTCGAAGCTTTACC	TTGTTGTGTGCCACAGCATAATGG	351	62.2	62.2
28	RM263	2	AATCTATGGACCTGGGAGGAACC	TGACGAGAGTGCTACGTTTGAGC	235	63.2	63.2
29	RM5470	2	CGTGTATTGCATGGATTGTCTGG	CGGAACCCACAATTTCTTTCTGC	269	62.1	60.6
30	RM523	3	TGAATTCTTGCACATGGTCAGC	TGGGAGGTTTGCTAGGGTAATCC	252	63.5	60.2
31	RM22	3	GTTGACAAACAGGCCCTGAAACC	GTATGGTCTGGAGACGACGATCC	171	64	64
32	A03P09039	3	CAAGGATAGGTGAGGGAGT	CCTGTGATCTTTACCGATCC	166	55.1	55.1
33	RM232	3	CCGGTATCCTTCGATATTGC	CCGACTTLTCCTCCLGACG	156	58.9	55
34	A03P23446	3	TCGAGAGTTGTTGTCGGGAT	TAGTAACCACAACCAAACCGT	206	58.9	58.3
35	A03P27107	3	GGCAAGGCAGCGACAATTC	TGGGAAGAATACTGGTCTGC	153	59.1	56.3
36	RM16097	3	CGCCTCGTAAGGTTGAGATCG	TGCCCTGTTCTTTCCATCTTGC	383	62.6	62.6
37	RM85	3	CCAAAGATGAAACCTGGATTG	GCACAAGGTGAGCAGTCC	103	58.2	57
38	A04P01363	4	GGTGTTTTTCATTTTGACTACCC	ACACACTAACGAAAATGATGAAG	157	55.8	55.8
39	A04P01756	4	GGACAATCAGTTAGTGGAC	GCTGTGGCAACTTGACATT	305	55.2	52.5
40	A04P08530	4	CGGCAAACTTATGAAGCCAC	AACGAGGGACAAAATGCCTG	316	57.9	56.4
41	A04P11418	4	GATTAAGGGAGCAGTGGGAA	GTTTTGGTGAACACGGCATG	117	57.5	56.7
42	A04P13832	4	ACGGGGAACTTGCTGAAT	GAGCACTTGTTGACTGGT	537	54.3	54.3
43	A04P13862	4	TTTTGCCGGATGATCAGA	CCCATCAAGAATTGCCAG	250	53.4	53.4

S. No	Marker	Chromosome	Forward (5′ to 3′)	Reverse (5′ to 3′)	Product size	Tm (°C)	Ta (°C)
44	RM471	4	AGAAATGGATCGGACTGAACATGC	AGACACTCGGACGCACAAGC	198	61.6	61.6
45	RM3367	4	GCCTCGGCTGATGTAACCTTCC	GATATGTGCTGCTGTGTGCTTGC	97	64.9	64.9
46	RM17201	4	GATCGTTGCTGCTTTCAATGAGG	AGTGTTTCACCTTGGACCCATGC	190	64.5	61.1
47	RM241	4	GTTCATTTCGTGATCTCTGAGC	GCAGATTTACAGGTTTGCTAGG	264	57.9	57.9
48	RM17296	4	GTTCATTTCGTGATCTCTGAGC	GCAGATTTACAGGGTTGCTAGG	264	56.7	56
49	RM127	4	CGAAGCTTTCGGTGGGATAGC	ACCTTGAGCGAGTCCTTGAACG	195	64.1	63.1
50	RM17728	5	CCTCGAGCATCATCATCAGTAGG	TCCTCTTCTTGCTTGCTTCTTCC	80	61.8	61.2
51	A05P00597	5	CACTATGCTAGAGACTGTCC	TTCCCATATGTCTGGTGCTG	197	56.3	53
52	A05P05283	5	CAACAGGGCAACCAACTTAC	GGCACGATGCATGACATACA	342	56.7	56.4
53	RM249	5	CAACTCCACTCCAGACTCAACTCC	GGTATGATGCCATGAAGGTCAGC	294	62.2	62.2
54	A05P10927	5	TATCCCCATCATGCAGAC	GTTCACAAATGTGAAGGGCG	208	57.1	53.1
55	A05P13060	5	ACGGCGGCATGAGATTGATT	AAAACCAGGCCAACCTATGG	271	57.5	57.5
56	RM164	5	TCTTGCCCGTCACTGCAGATATCC	GCAGCCCTAATGCTACAATTCTTC		60	60
57	A05P22287	5	GGACCATCTCCCGTTTACT	GAATGGGCTATGGGTCACTT	143	56.5	56.5
58	A05P25260	5	GCCTCTTAGCATTTAGCAGC	CCAGGGAACGCATGAGCT	196	59.5	54.7
59	A05P26105	5	CATACATTTGACGCATGCAGC	CTGCACAACACAAAGCACTC	147	56.4	56.4
60	RM586	6	TGCCATCTCATAAACCCACTAACC	CTGAGATACGCCAACGAGATACC	295	61.5	61.3
61	RM3408	6	AGTGAATCCCATCAGATCACTCC	CATATCAAATCGCCGAGAAGG	211	58.3	58.3
62	A06P08188	6	AGAGGAGCTGATTTGTTTGGT	CTGTCGGCGCAGATCTTTC	170	58.3	58.3
63	A06P22133	6	CAAGAATTCAGGCGGTGTAC	CCTTTACCAAGACATTGGCAC	210	58.9	55.9
64	RM20818	7	AGATGCAGATAGATGCATGTCACG	ACCGATCATCCACGATCCTACG	375	63.3	60.3
65	RM481	7	TAGCTAGCCGATTGAATGGC	CTCCACCTCCTATGTTGTTG	169	54.9	54.9
66	RM21097	7	TCCGGTCGTCCTCATCGTATCC	GCCCTCTTGCTCCCACATCG	185	63	63
67	RM21577	7	GCCGAAAGTAGCTCGTCAATGG	GCTCAATGAGTCCTCTGTTGTGG	275	62.5	62.5
68	RM21652	7	CTTTCTGTCTCACGCTGGATTGG	TGCGTACGTGATGAATGGTTAGC	186	62.8	61.4
69	RM1132	7	TCAAGGTCGACATGTTAGGTATGC	AACCCTATCACCTGAGAAACATCC	88	61.2	60.9
70	RM21976	7	CTTCCTCCTACCTTCCTCCATCC	GCACCATCACCTCCATCTCTAGC	195	63.7	63.7
71	RM22554	8	TTGTCAAGATCATCCTCGTAGC	GTCATTCTGCAACCTGAGATCC	283	60.4	58.9
72	A08P23255	8	TGACACATCTAGTGAAGCACA	TCACCGCATAGAGTAAGAACA	191	57.5	57.5
73	A08P25335	8	TGATCCCTACAAATGGTCGTT	TGCTACCTTGCATATGGGC	138	58.6	56.1

S. No	Marker	Chromosome	Forward (5′ to 3′)	Reverse (5′ to 3′)	Product size	Tm (°C)	Ta (°C)
74	RM23556	8	ACCATGATGGGCTAATGACG	TTCATCAGATATCAGCGACAGG	89	58.5	56.5
75	RM23914	9	GAGGATCCTTACCATCAAACTTCG	CCAAGAACCTGCATTCTTCAAGG	197	61.2	60.4
76	A09P07331	9	CTTCTGCCCACCATTTTTGG	TGGAGAACAGCACCAATCGA	236	58.5	57.1
77	A09P09438	9	CTCGTGTATCATATGCATAGG	TGTTTCTTGCCTGTCCTTTTG	348	58.7	53.8
78	RM13021	9	GATTCCACGTCAGGATCTTCTGG	GCTCACCAGTTGAGATTGAAAGG	196	60.9	60.9
79	A09P12377	9	GTACATGGCGAATACCAAGAC	GAACTCCGAGATCAGGTCC	145	57.6	57.6
80	A09P18034	9	CTTGATGCGTTGGCAGGTAA	GAAAACATGGAGCTCGGTCA	219	57.6	57.3
81	RM3808	9	CAGTGGCGTGGAGAGAAATTTGG	CTCACCTGCGACAGCAAGATCG	145	64.7	63.4
82	RM216	10	GATGGTAAAGGAAGAACGTGTGC	CACTCATAGACGCATCACATAGCC	91	61.1	61
83	A10P05672	10	CATGGCATTTTAAGCTCGCC	TTGTCTAGTTGCCAGACCCA	260	57.9	56.4
84	A10P09104	10	GCAAAGTTTACCCTCTAGCG	GTGTGGTGCAACCTCAGGT	147	59.7	55.3
85	RM25280	10	TAGGACTCGATCAAGGGAATTGG	TTCCTTCCTTTCTCTCCCTTTACC	250	61.7	61.3
86	RM25271	10	AGACGCTACTCCCACCTGTAACC	ATATCATTGCCGCAACACAAGC	186	60	60
87	RM25626	10	ATGCTCTCAAGTGTGTCAAGG	AACCTCTGGAGTATGTGTAGTGC	399	60.2	59.2
88	RM6737	10	GCACGTAAATGATAGGCACCATTGC	CACAAGGTGGTGTGGGCTAGACG	393	66.8	62.4
89	RM6100	10	TTCCCTGCAAGATTCTAGCTACACC	TGTTCGTCGACCAAGAACTCAGG	173	64.1	62.5
90	RM25794	10	CTTCTCCGTGCATCTAGGAATGG	GGGAGCTTCATCGTTTCTTTAAGACC	197	62.6	62
91	A10P22154	10	TGGCTCATATGTTCTCTGGC	CGCAAGTTCAGTAAGGTCCA	149	57	56
92	RM12568	11	TCTTCGAAACAAGCAGCTCTAGG	GATAGGTCGGATGTGTGATTTGG	187	60.4	60.4
93	A11P05095	11	GTCCAGGCATAACTGAAACC	AGCCAATTCAAAGCAGCCGA	172	59	55.8
94	RM287	11	GGCTACACCTACACGCGAGAACC	AGATGCATGGAATGCCTGTTTGG	299	62.2	62.2
95	RM13347	11	TCCTTCTCCTTGAACAGCGACAGC	AGAGGCGAGAGGCATGGAGTGC	171	67.7	65.6
96	RM206	11	ATCGATCCGTATGGGTTCTAGC	GTCCATGTAGCCAATCTTATGTGG	477	60.6	59.8
97	A11P25204	11	AGGGGAAAGGCGAAATGGCA	TGCGAATGATCCATGTGTACA	142	61.8	58.5
98	RM224	11	ATCGATCGATCTTCACGAGG	TGCTATAAAAGGCATTCGGG	157	56.3	54.5
99	A12P02180	12	CTGGAGAATTGCTCGATTCC	TCACAAACAGAACATATCACAC	166	55.3	55.3
100	RM27564	12	CTTCTTCGAGGGTTTCTCGTTGC	CTCCTCTTATGCCCACCACTTCC	84	64.4	63.3
101	A12P05053	12	CAAATCTTTGACATGCACCTTG	GAGCTAAAGTCCCAACGTTG	168	57.4	56
102	A12P22450	12	TCCGTGGACACGCTTCCTA	TCTACTTGTAACACGATCAAAG	312	60.6	54.7
103	RM235	12	AAGCTAGGGCTAACGAACGAACG	TCTCCATCTCCATCTCCATCTCC	157	63.4	62.7

Table S3. Details of Di-Genic epistatic QTLs detected for GPC, grain quality and yield traits in F_2 population

S. No	ID	Trait	Chr 1	Position 1	Left Marker 1	Right Marker 1	Chr 2	Position 2	Left Marker 2	Right Marker 2	LOD	PVE	Add1	Add2	Add by Add
1	1	PC	1	170	RM562	RM11307	1	235	RM11307	RM10890	7.6224	4.306	1.6446	0.0763	-1.3891
2	1	PC	1	315	RM10890	RM128	2	215	RM263	RM5470	6.6568	3.4275	-0.4235	1.7611	-0.7153
3	1	PC	1	315	RM10890	RM128	3	130	A03P27107	RM85	6.0801	3.8331	1.1188	0.8299	-0.2432
4	1	PC	4	95	RM471	RM17201	4	170	RM241	RM127	5.6514	3.7013	1.6056	-0.6771	-0.8685
5	1	PC	1	275	RM11307	RM10890	4	180	RM241	RM127	5.8399	2.9263	0.4302	1.1165	-1.4092
6	1	PC	3	85	RM232	A03P23446	5	20	A05P00597	A05P05283	5.1658	2.9165	-0.2537	0.9537	-0.0846
7	1	PC	1	315	RM10890	RM128	5	80	A05P10927	RM164	5.4879	3.0026	0.2651	0.3137	-1.0854
8	1	PC	1	165	RM562	RM11307	6	0	A06P22133	A06P08188	6.7755	3.6192	0.28	1.3827	0.0659
9	1	PC	2	85	A02P10359	RM6165	7	20	RM20818	RM481	5.1865	2.6612	-0.4848	1.4047	-0.2105
10	1	PC	3	150	A03P27107	RM85	7	150	RM21652	RM1132	5.2176	2.8434	0.7782	-0.32	-0.486
11	1	PC	1	390	RM128	RM1297	7	155	RM21652	RM1132	6.8675	3.0848	1.1947	-0.1846	-0.409
12	1	PC	3	85	RM232	A03P23446	8	25	RM23556	A08P25335	5.1278	2.6446	0.5717	0.3868	0.4711
13	1	PC	1	310	RM10890	RM128	8	60	A08P23255	RM22554	6.4487	3.2009	0.9558	0.7076	-0.8554
14	1	PC	2	205	RM263	RM5470	9	65	RM13021	A09P12377	5.3649	3.9374	1.1131	1.2513	0.9187
15	1	PC	1	320	RM10890	RM128	9	70	RM13021	A09P12377	6.9943	4.1919	-0.5353	1.8041	-0.8677
16	1	PC	7	175	RM1132	RM21976	11	65	A11P05095	RM12568	5.2874	2.788	-0.5243	0.821	-0.1812
17	1	PC	4	90	RM471	RM17201	11	75	A11P05095	RM12568	6.2243	3.9502	1.3576	-0.8976	-1.1478
18	1	PC	2	95	A02P10359	RM6165	11	190	RM13347	RM206	5.0369	3.0041	0.9851	-0.0148	-1.5584
19	1	PC	8	20	RM23556	A08P25335	11	190	RM13347	RM206	5.1915	3.5986	0.5487	0.8616	0.2942
20	1	PC	11	5	RM287	A11P05095	11	200	RM13347	RM206	5.2097	2.6358	0.2876	1.1918	-0.2494
21	1	PC	10	60	RM25280	RM6737	11	205	RM13347	RM206	5.8144	3.5863	0.2524	-0.3596	-2.1383
22	1	PC	1	320	RM10890	RM128	11	240	RM206	A11P25204	6.3329	3.8313	1.5831	-0.1307	-0.2581
23	1	PC	1	165	RM562	RM11307	12	90	RM27564	A12P02180	6.871	3.766	1.4415	0.9282	-0.5934
24	1	PC	11	60	A11P05095	RM12568	12	115	A12P02180	RM235	5.0856	3.5542	0.6818	1.7934	0.1847
25	1	PC	9	75	RM13021	A09P12377	12	120	A12P02180	RM235	6.8783	4.2603	0.4654	2.2167	0.651

S. No	ID	Trait	Chr 1	Position 1	Left Marker 1	Right Marker 1	Chr 2	Position 2	Left Marker 2	Right Marker 2	LOD	PVE	Add1	Add2	Add by Add
26	1	PC	12	10	A12P22450	A12P05053	12	120	A12P02180	RM235	5.0745	3.48	0.7598	-0.5244	-0.8757
27	1	PC	4	40	A04P13832	RM471	12	125	A12P02180	RM235	6.2867	3.0703	0.8154	1.2409	-0.1299
28	1	PC	6	0	A06P22133	A06P08188	12	145	A12P02180	RM235	5.0293	3.1523	0.6188	0.9795	0.6127
29	2	AC	1	340	RM10890	RM128	1	350	RM10890	RM128	14.5348	1.3816	2.7086	-2.4983	4.7411
30	2	AC	1	160	RM562	RM11307	2	10	A02P01132	A02P03360	11.6947	1.3485	-1.7017	0.4893	0.4474
31	2	AC	2	5	A02P01132	A02P03360	2	25	A02P03360	A02P07438	12.3234	1.2122	-1.2367	0.3685	0.8045
32	2	AC	3	5	RM523	RM22	3	20	RM22	A03P09039	13.5174	1.2505	0.2945	-0.4283	1.7012
33	2	AC	2	215	RM263	RM5470	3	130	A03P27107	RM85	11.6181	1.2937	2.6376	-2.888	2.3719
34	2	AC	1	5	RM6120	RM3233	3	140	A03P27107	RM85	14.8844	1.3274	2.9041	-2.6459	2.6003
35	2	AC	4	100	RM471	RM17201	4	105	RM471	RM17201	11.9291	1.181	-0.4316	0.1153	0.0067
36	2	AC	1	135	RM562	RM11307	4	110	RM17201	RM3367	12.3997	1.1788	4.8592	-1.2118	0.9736
37	2	AC	3	205	RM85	RM16097	4	145	RM3367	RM17296	11.4308	1.2406	0.8527	-1.2249	3.843
38	2	AC	2	210	RM263	RM5470	4	180	RM241	RM127	11.2279	1.4038	3.5186	-2.5567	2.0516
39	2	AC	1	135	RM562	RM11307	5	30	A05P00597	A05P05283	11.0679	1.1894	2.5394	-2.9275	2.4041
40	2	AC	2	25	A02P03360	A02P07438	5	30	A05P00597	A05P05283	11.3118	1.2448	1.8105	-2.1669	1.8082
41	2	AC	4	170	RM241	RM127	5	30	A05P00597	A05P05283	8.1763	1.2255	0.8265	-3.9546	1.0643
42	2	AC	3	130	A03P27107	RM85	5	115	A05P22287	A05P26105	10.3661	1.3015	-2.1518	-3.4843	-0.8191
43	2	AC	5	120	A05P22287	A05P26105	5	125	A05P22287	A05P26105	11.4929	1.0862	0.0883	-0.339	-0.0191
44	2	AC	2	30	A02P03360	A02P07438	6	10	A06P22133	A06P08188	9.437	1.1934	3.9776	1.4032	-0.9964
45	2	AC	6	20	A06P22133	A06P08188	6	25	A06P08188	RM3408	13.1004	1.168	-0.7841	1.0889	0.9302
46	2	AC	4	180	RM241	RM127	6	30	A06P08188	RM3408	7.8414	1.1488	-3.0557	3.3919	2.6097
47	2	AC	3	125	A03P27107	RM85	6	55	RM3408	RM586	10.338	1.1234	-1.7782	3.464	2.0383
48	2	AC	5	25	A05P00597	A05P05283	6	60	RM3408	RM586	8.539	1.202	-2.7397	2.0826	1.3149
49	2	AC	1	130	RM562	RM11307	6	65	RM3408	RM586	11.5539	1.2438	1.64	3.1762	-2.2062
50	2	AC	4	175	RM241	RM127	7	70	RM481	RM21097	11.3778	1.2592	-2.1232	-1.0323	-5.2308
51	2	AC	3	155	A03P27107	RM85	7	115	RM21577	RM21652	11.0523	1.2933	-1.406	-1.3484	-4.3413
52	2	AC	1	160	RM562	RM11307	7	120	RM21577	RM21652	13.3022	1.3036	4.1459	-1.3955	0.9604
53	2	AC	5	30	A05P00597	A05P05283	7	120	RM21577	RM21652	11.6429	1.1148	-2.02	2.0695	2.3487
54	2	AC	7	135	RM21577	RM21652	7	140	RM21652	RM1132	15.3253	1.3119	-1.1517	1.037	2.9225
55	2	AC	2	25	A02P03360	A02P07438	7	145	RM21652	RM1132	10.0957	1.1817	-0.3861	0.3654	0.2789

S. No	ID	Trait	Chr 1	Position 1	Left Marker 1	Right Marker 1	Chr 2	Position 2	Left Marker 2	Right Marker 2	LOD	PVE	Add1	Add2	Add by Add
56	2	AC	6	60	RM3408	RM586	7	155	RM21652	RM1132	10.1261	1.2178	2.5654	2.3559	-2.0193
57	2	AC	6	60	RM3408	RM586	8	5	RM23556	A08P25335	9.9752	1.2009	2.1273	2.4187	-1.5427
58	2	AC	2	25	A02P03360	A02P07438	8	15	RM23556	A08P25335	9.2613	1.0931	1.4915	2.0459	-2.3982
59	2	AC	3	140	A03P27107	RM85	8	15	RM23556	A08P25335	11.5427	1.2612	-1.9297	3.32	1.4867
60	2	AC	5	30	A05P00597	A05P05283	8	15	RM23556	A08P25335	8.1685	1.126	-2.1213	2.1631	1.9256
61	2	AC	8	20	RM23556	A08P25335	8	40	A08P25335	A08P23255	15.6339	1.3326	-0.869	0.8187	5.2508
62	2	AC	7	120	RM21577	RM21652	8	45	A08P25335	A08P23255	11.8844	1.2751	-0.8817	1.1516	4.1098
63	2	AC	1	145	RM562	RM11307	8	70	A08P23255	RM22554	11.1427	1.5166	5.7451	1.0729	-1.3149
64	2	AC	4	95	RM471	RM17201	8	75	A08P23255	RM22554	11.6204	1.2532	-1.7952	1.0038	5.2316
65	2	AC	1	10	RM6120	RM3233	9	30	RM13021	A09P12377	12.7014	1.3185	2.853	-1.8435	2.7549
66	2	AC	5	120	A05P22287	A05P26105	9	40	RM13021	A09P12377	10.5439	1.1365	-0.1904	0.2539	0.0143
67	2	AC	7	125	RM21577	RM21652	9	40	RM13021	A09P12377	12.6885	1.3337	-1.4126	-1.2135	-4.3689
68	2	AC	3	20	RM22	A03P09039	9	65	RM13021	A09P12377	11.964	1.3	2.3299	-2.6377	1.1182
69	2	AC	4	195	RM241	RM127	9	80	RM13021	A09P12377	7.9826	1.1142	-2.7141	-2.4507	-2.706
70	2	AC	8	20	RM23556	A08P25335	9	85	RM13021	A09P12377	14.1411	1.3043	2.8669	-1.5985	2.1
71	2	AC	2	35	A02P03360	A02P07438	9	90	RM13021	A09P12377	11.8884	1.2255	2.5771	-1.9725	1.3779
72	2	AC	9	40	RM13021	A09P12377	9	110	A09P12377	RM23914	14.2593	1.6639	1.7682	-1.0204	3.8903
73	2	AC	6	55	RM3408	RM586	9	135	A09P09438	A09P18034	10.215	1.188	3.0321	-1.9627	2.7581
74	2	AC	5	30	A05P00597	A05P05283	10	50	RM25280	RM6737	9.8216	1.3428	-3.1201	0.5947	2.288
75	2	AC	6	45	RM3408	RM586	10	55	RM25280	RM6737	7.466	1.1138	2.9794	2.0693	-2.5011
76	2	AC	9	25	RM13021	A09P12377	10	100	RM6737	RM25626	11.428	1.3336	-2.1102	-1.979	-3.6599
77	2	AC	8	10	RM23556	A08P25335	10	110	RM25626	RM6100	8.5718	1.0262	2.9224	-2.0905	2.0805
78	2	AC	4	100	RM471	RM17201	10	120	RM25626	RM6100	10.9862	1.1943	-1.3009	-1.206	-5.2178
79	2	AC	7	120	RM21577	RM21652	10	120	RM25626	RM6100	9.1604	0.9495	-2.7138	2.6017	2.1892
80	2	AC	3	135	A03P27107	RM85	10	125	RM25626	RM6100	10.4774	1.0455	-2.4232	2.331	2.4463
81	2	AC	10	120	RM25626	RM6100	10	135	RM6100	RM25794	11.9688	1.054	-1.4992	1.7396	2.4079
82	2	AC	1	130	RM562	RM11307	10	155	RM6100	RM25794	10.7703	1.2051	3.0828	-1.3088	2.6358
83	2	AC	2	35	A02P03360	A02P07438	10	155	RM6100	RM25794	9.8865	1.195	1.8181	2.9118	-1.9769
84	2	AC	8	10	RM23556	A08P25335	11	20	RM287	A11P05095	10.2536	1.0368	2.183	-1.7686	2.5174
85	2	AC	3	135	A03P27107	RM85	11	80	A11P05095	RM12568	12.7396	1.4455	-1.5192	4.2646	1.4373

S. No	ID	Trait	Chr 1	Position 1	Left Marker 1	Right Marker 1	Chr 2	Position 2	Left Marker 2	Right Marker 2	LOD	PVE	Add1	Add2	Add by Add
86	2	AC	2	20	A02P03360	A02P07438	11	85	A11P05095	RM12568	11.704	1.2465	2.763	-2.277	1.5351
87	2	AC	4	125	RM17201	RM3367	11	85	A11P05095	RM12568	12.8911	1.3047	-1.4016	0.8141	5.2159
88	2	AC	7	155	RM21652	RM1132	11	85	A11P05095	RM12568	12.8366	1.3438	-1.8411	1.1742	4.3944
89	2	AC	9	85	RM13021	A09P12377	11	190	RM13347	RM206	12.645	1.2058	-1.2776	1.8615	3.3549
90	2	AC	10	115	RM25626	RM6100	11	200	RM13347	RM206	11.0499	1.1017	-1.834	1.7388	3.4049
91	2	AC	5	45	A05P05283	A05P13060	11	205	RM13347	RM206	10.2084	1.3807	-1.4589	-1.4756	-0.389
92	2	AC	11	205	RM13347	RM206	11	220	RM13347	RM206	12.7111	1.3793	0.749	-0.7893	5.2998
93	2	AC	6	60	RM3408	RM586	11	240	RM206	A11P25204	10.3967	1.3433	1.9079	-1.0287	3.4498
94	2	AC	1	125	RM562	RM11307	11	245	RM206	A11P25204	13.863	1.5839	2.9478	3.0303	-3.0989
95	2	AC	11	190	RM13347	RM206	12	15	A12P22450	A12P05053	10.7707	1.1974	2.2585	-3.2012	1.3082
96	2	AC	6	50	RM3408	RM586	12	20	A12P22450	A12P05053	10.7099	1.2044	2.9435	-2.6071	2.0753
97	2	AC	4	180	RM241	RM127	12	85	RM27564	A12P02180	10.5359	1.1952	-3.1353	2.8057	2.95
98	2	AC	9	85	RM13021	A09P12377	12	110	A12P02180	RM235	10.7389	1.1102	0.2608	-0.4221	-0.2048
99	2	AC	12	105	A12P02180	RM235	12	115	A12P02180	RM235	12.4142	1.0849	0.0997	-0.2181	0.9488
100	2	AC	1	120	RM562	RM11307	12	135	A12P02180	RM235	11.4648	1.5278	-1.4554	1.2954	6.7092
101	2	AC	7	115	RM21577	RM21652	12	135	A12P02180	RM235	12.8238	1.262	-1.484	0.8583	4.2139
102	2	AC	8	15	RM23556	A08P25335	12	135	A12P02180	RM235	10.4415	1.1979	2.4943	-1.9751	1.5426
103	2	AC	10	45	RM25280	RM6737	12	135	A12P02180	RM235	10.409	1.2677	2.5848	2.153	-2.7854
104	2	AC	2	25	A02P03360	A02P07438	12	140	A12P02180	RM235	10.215	1.2666	3.5148	1.0262	-1.5573
105	2	AC	3	135	A03P27107	RM85	12	150	A12P02180	RM235	13.1405	1.3391	-1.7686	1.0907	3.9473
106	2	AC	5	115	A05P22287	A05P26105	12	150	A12P02180	RM235	10.9311	1.2496	-3.1435	2.5719	2.7056
107	3	GC	1	335	RM10890	RM128	1	340	RM10890	RM128	27.2231	1.4398	9.2596	-2.4079	-26.9943
108	3	GC	1	385	RM128	RM1297	2	195	RM263	RM5470	22.9779	1.3444	-13.4503	-13.842	16.8725
109	3	GC	2	205	RM263	RM5470	2	210	RM263	RM5470	26.3224	1.3601	-2.1239	9.4848	-24.4117
110	3	GC	3	20	RM22	A03P09039	3	80	RM232	A03P23446	18.7613	1.0569	7.7023	-7.2421	-6.3692
111	3	GC	1	310	RM10890	RM128	3	135	A03P27107	RM85	31.9499	1.4627	11.353	-2.6988	-11.9263
112	3	GC	2	160	RM341	RM13601	3	150	A03P27107	RM85	23.0397	1.4345	6.0667	-9.0937	-6.3408
113	3	GC	2	200	RM263	RM5470	4	75	RM471	RM17201	16.4691	1.0314	-2.0993	0.1127	3.2273
114	3	GC	1	385	RM128	RM1297	4	80	RM471	RM17201	22.8448	1.421	-6.6406	6.8045	-16.4087
115	3	GC	3	145	A03P27107	RM85	4	80	RM471	RM17201	27.2902	1.4424	21.163	-14.8407	-14.4168

S. No	ID	Trait	Chr 1	Position 1	Left Marker 1	Right Marker 1	Chr 2	Position 2	Left Marker 2	Right Marker 2	LOD	PVE	Add1	Add2	Add by Add
116	3	GC	4	75	RM471	RM17201	4	180	RM241	RM127	17.3086	0.9636	-13.3477	-10.0934	12.3819
117	3	GC	3	195	RM85	RM16097	5	20	A05P00597	A05P05283	17.4333	1.3243	-13.4493	16.9163	-10.0783
118	3	GC	2	200	RM263	RM5470	5	30	A05P00597	A05P05283	23.9137	1.4195	5.4349	-5.1641	-31.5907
119	3	GC	1	250	RM11307	RM10890	5	70	A05P10927	RM164	22.2134	1.3498	15.797	14.7692	15.167
120	3	GC	4	180	RM241	RM127	5	70	A05P10927	RM164	17.2361	0.9636	-8.6861	18.6871	-11.9811
121	3	GC	5	115	A05P22287	A05P26105	5	125	A05P22287	A05P26105	19.0551	0.9399	4.8458	-3.1337	-0.7946
122	3	GC	1	145	RM562	RM11307	6	5	A06P22133	A06P08188	25.274	1.4241	-7.1257	15.7685	-7.0757
123	3	GC	2	200	RM263	RM5470	6	10	A06P22133	A06P08188	17.7255	0.9495	2.8293	-2.6033	-2.9307
124	3	GC	4	175	RM241	RM127	6	10	A06P22133	A06P08188	17.7519	0.9159	-16.1368	11.8246	-13.3847
125	3	GC	6	10	A06P22133	A06P08188	6	30	A06P08188	RM3408	20.7538	0.9579	-3.5891	1.6749	2.3213
126	3	GC	3	150	A03P27107	RM85	6	60	RM3408	RM586	24.0671	1.3849	22.3715	14.1329	14.343
127	3	GC	5	70	A05P10927	RM164	6	60	RM3408	RM586	14.8733	0.9122	16.0156	-10.9764	-12.0183
128	3	GC	1	310	RM10890	RM128	7	20	RM20818	RM481	24.6662	1.3694	18.4529	-12.3777	-12.8397
129	3	GC	3	145	A03P27107	RM85	7	20	RM20818	RM481	19.7594	1.3339	19.2216	-11.5133	-12.3378
130	3	GC	4	175	RM241	RM127	7	20	RM20818	RM481	20.2965	1.3204	-21.051	-9.268	10.8298
131	3	GC	6	10	A06P22133	A06P08188	7	20	RM20818	RM481	21.4633	1.2911	18.5168	-10.89	-10.5699
132	3	GC	5	25	A05P00597	A05P05283	7	25	RM20818	RM481	17.9507	0.9744	8.679	-11.7714	-14.7725
133	3	GC	7	120	RM21577	RM21652	7	140	RM21652	RM1132	20.9348	0.9212	15.1825	-15.1696	-12.9438
134	3	GC	2	205	RM263	RM5470	7	150	RM21652	RM1132	21.1251	1.3631	15.6447	-14.7807	-15.4712
135	3	GC	3	145	A03P27107	RM85	8	10	RM23556	A08P25335	18.574	1.1065	11.5792	-22.0881	-8.7296
136	3	GC	1	235	RM11307	RM10890	8	15	RM23556	A08P25335	19.4414	1.3504	21.6991	10.0372	9.868
137	3	GC	2	195	RM263	RM5470	8	15	RM23556	A08P25335	18.3651	1.0186	-8.9313	10.0361	-20.3418
138	3	GC	4	180	RM241	RM127	8	15	RM23556	A08P25335	17.6166	0.9441	-13.6427	-7.766	16.2797
139	3	GC	6	60	RM3408	RM586	8	15	RM23556	A08P25335	16.6456	0.9471	6.5971	-25.222	-5.389
140	3	GC	7	25	RM20818	RM481	8	20	RM23556	A08P25335	15.6361	0.9773	-12.1275	9.0857	-13.9681
141	3	GC	8	10	RM23556	A08P25335	8	25	RM23556	A08P25335	20.1073	0.9529	1.4235	-0.3087	-6.0251
142	3	GC	5	70	A05P10927	RM164	8	75	A08P23255	RM22554	14.0182	0.9797	6.6708	-5.0016	-4.6813
143	3	GC	2	80	A02P10359	RM6165	9	25	RM13021	A09P12377	23.7613	1.4007	5.7656	-6.2199	-19.92
144	3	GC	6	60	RM3408	RM586	9	25	RM13021	A09P12377	25.5737	1.3866	-7.6735	-7.5146	7.3989
145	3	GC	4	180	RM241	RM127	9	35	RM13021	A09P12377	20.6274	1.3524	-15.5563	15.6485	-15.735

S. No	ID	Trait	Chr 1	Position 1	Left Marker 1	Right Marker 1	Chr 2	Position 2	Left Marker 2	Right Marker 2	LOD	PVE	Add1	Add2	Add by Add
146	3	GC	5	130	A05P26105	A05P25260	9	35	RM13021	A09P12377	18.6989	1.3428	4.0384	-4.8163	3.8277
147	3	GC	7	20	RM20818	RM481	9	35	RM13021	A09P12377	21.5734	1.3551	-15.1934	14.1148	-8.3018
148	3	GC	8	10	RM23556	A08P25335	9	35	RM13021	A09P12377	23.1733	1.3577	-14.5281	15.2908	-15.6174
149	3	GC	3	140	A03P27107	RM85	9	75	RM13021	A09P12377	31.5502	1.4796	11.4021	24.9423	1.4783
150	3	GC	1	240	RM11307	RM10890	9	80	RM13021	A09P12377	27.137	1.4288	-2.6685	25.4841	-10.4797
151	3	GC	9	25	RM13021	A09P12377	9	150	A09P09438	A09P18034	27.5211	1.4452	10.4866	25.1208	2.4401
152	3	GC	1	305	RM10890	RM128	10	55	RM25280	RM6737	21.8526	1.3629	3.4333	3.8432	-5.9839
153	3	GC	5	25	A05P00597	A05P05283	10	60	RM25280	RM6737	16.2952	1.1653	14.7379	-15.3094	-11.4141
154	3	GC	8	75	A08P23255	RM22554	10	60	RM25280	RM6737	20.3473	1.3452	9.7451	-19.9645	-10.4268
155	3	GC	7	20	RM20818	RM481	10	70	RM25280	RM6737	19.2111	1.3454	-3.6265	2.6216	2.5377
156	3	GC	3	195	RM85	RM16097	10	75	RM25280	RM6737	21.1327	1.3481	3.3088	-2.8114	-3.3674
157	3	GC	10	75	RM25280	RM6737	10	85	RM25280	RM6737	18.6224	0.9183	-13.3206	11.8912	-14.3537
158	3	GC	4	75	RM471	RM17201	10	125	RM25626	RM6100	19.5038	0.915	-15.5639	-12.4996	10.9246
159	3	GC	6	10	A06P22133	A06P08188	10	150	RM6100	RM25794	19.9489	0.9348	14.3788	-12.957	-14.7627
160	3	GC	9	35	RM13021	A09P12377	10	150	RM6100	RM25794	23.1615	1.3594	15.4059	-7.8588	-15.225
161	3	GC	2	200	RM263	RM5470	10	155	RM6100	RM25794	18.787	1.3577	4.0937	-3.2989	-3.3361
162	3	GC	1	490	RM11996	RM1067	11	20	RM287	A11P05095	26.3974	1.4679	8.8013	-4.0791	-27.4213
163	3	GC	10	150	RM6100	RM25794	11	60	A11P05095	RM12568	23.2734	1.3628	-9.9228	12.4374	-18.2767
164	3	GC	3	200	RM85	RM16097	11	85	A11P05095	RM12568	28.3673	1.4287	5.8346	-6.0555	-23.9496
165	3	GC	8	15	RM23556	A08P25335	11	185	RM13347	RM206	18.1469	1.3414	-16.8691	8.0368	-6.5269
166	3	GC	2	190	RM263	RM5470	11	200	RM13347	RM206	27.458	1.4209	-6.3425	6.6215	-29.3686
167	3	GC	6	55	RM3408	RM586	11	200	RM13347	RM206	25.7424	1.4109	2.251	-9.689	-27.1102
168	3	GC	9	20	RM13021	A09P12377	11	200	RM13347	RM206	28.1407	1.4372	5.7433	-6.0335	-30.8846
169	3	GC	11	75	A11P05095	RM12568	11	200	RM13347	RM206	23.8758	1.3815	30.7986	-5.6633	-6.1548
170	3	GC	4	45	A04P13832	RM471	11	235	RM206	A11P25204	18.1655	0.9972	1.6915	2.9075	-26.1945
171	3	GC	5	25	A05P00597	A05P05283	11	240	RM206	A11P25204	22.1607	1.3764	16.3367	9.3568	9.8806
172	3	GC	7	20	RM20818	RM481	11	245	RM206	A11P25204	23.2996	1.4286	-22.8249	-6.2021	1.7943
173	3	GC	7	20	RM20818	RM481	12	60	A12P05053	RM27564	16.0157	1.0883	-28.1072	1.702	-3.0859
174	3	GC	9	35	RM13021	A09P12377	12	60	A12P05053	RM27564	20.9326	1.3884	21.445	13.7219	14.1238
175	3	GC	11	75	A11P05095	RM12568	12	60	A12P05053	RM27564	21.8524	1.3763	0.958	9.1293	-26.414

S. No	ID	Trait	Chr 1	Position 1	Left Marker 1	Right Marker 1	Chr 2	Position 2	Left Marker 2	Right Marker 2	LOD	PVE	Add1	Add2	Add by Add
176	3	GC	2	30	A02P03360	A02P07438	12	85	RM27564	A12P02180	22.1153	1.3953	-0.2226	9.9513	-24.6962
177	3	GC	8	15	RM23556	A08P25335	12	125	A12P02180	RM235	19.6722	1.3777	3.9574	-3.4811	6.8475
178	3	GC	4	70	RM471	RM17201	12	135	A12P02180	RM235	17.0705	1.2993	-2.9524	3.3286	-5.405
179	3	GC	6	15	A06P22133	A06P08188	12	135	A12P02180	RM235	19.5232	1.3323	10.1406	20.2873	10.4162
180	3	GC	1	485	RM11996	RM1067	12	140	A12P02180	RM235	27.6965	1.4447	-5.1525	13.6482	-9.7978
181	3	GC	3	85	RM232	A03P23446	12	140	A12P02180	RM235	20.5988	1.325	-14.1632	14.7687	-15.5554
182	3	GC	10	165	RM6100	RM25794	12	140	A12P02180	RM235	22.1416	1.3492	-14.8918	15.5183	-15.1569
183	3	GC	12	20	A12P22450	A12P05053	12	150	A12P02180	RM235	18.8719	1.3548	8.1538	1.4967	1.5134
184	3	GC	5	115	A05P22287	A05P26105	12	170	A12P02180	RM235	18.6147	0.9893	-9.797	12.2039	-9.1792
185	4	KL	3	20	RM22	A03P09039	4	30	A04P08530	A04P13862	5.6231	3.175	0.0917	0.0859	-0.1195
186	4	KL	1	365	RM128	RM1297	7	165	RM21652	RM1132	5.676	4.9486	0.0491	0.0861	-0.0699
187	4	KL	3	90	RM232	A03P23446	7	165	RM21652	RM1132	5.0982	4.2018	0.0549	0.0748	-0.0616
188	4	KL	4	45	A04P13832	RM471	11	125	RM12568	RM13347	5.0221	6.6358	0.1531	0.1226	-0.1275
189	4	KL	2	100	RM6165	RM341	11	225	RM13347	RM206	5.2116	2.7705	0.0355	0.0905	-0.0673
190	5	KB	1	145	RM562	RM11307	1	495	RM11996	RM1067	6.2371	5.4695	0.128	0.0704	-0.0292
191	5	KB	3	40	RM22	A03P09039	3	205	RM85	RM16097	5.2276	4.077	0.0084	0.0024	-0.1007
192	5	KB	3	10	RM523	RM22	4	110	RM17201	RM3367	5.1585	1.1403	0.0243	0.0591	-0.0109
193	5	KB	1	155	RM562	RM11307	4	160	RM241	RM127	5.3744	5.0603	0.1031	0.07	-0.0737
194	5	KB	3	195	RM85	RM16097	9	25	RM13021	A09P12377	6.1432	5.529	-0.0224	0.0143	-0.0626
195	5	KB	9	40	RM13021	A09P12377	10	15	A10P09104	RM25271	5.1419	4.5596	0.0767	0.0596	-0.0556
196	5	KB	3	145	A03P27107	RM85	10	170	RM25794	A10P22154	5.0442	3.4521	-0.0006	0.0748	0.0034
197	5	KB	9	20	RM13021	A09P12377	11	70	A11P05095	RM12568	5.3844	5.3262	0.0369	0.1229	0.0634
198	5	KB	1	405	RM128	RM1297	11	140	RM12568	RM13347	5.3474	4.7279	0.0018	0.0346	0.0874
199	5	KB	4	90	RM471	RM17201	12	20	A12P22450	A12P05053	5.843	4.096	0.094	0.021	0.0211
200	5	KB	1	145	RM562	RM11307	12	50	A12P05053	RM27564	7.4136	4.7049	0.1098	0.0594	-0.033
201	5	KB	7	60	RM481	RM21097	12	85	RM27564	A12P02180	5.0962	3.6651	-0.0008	0.0203	0.0468
202	5	KB	11	115	RM12568	RM13347	12	135	A12P02180	RM235	7.8962	5.2173	0.068	0.077	-0.028
203	5	KB	2	25	A02P03360	A02P07438	12	145	A12P02180	RM235	5.2464	4.6211	0.0849	0.0775	0.0289
204	6	LB	1	530	RM1067	RM12241	4	5	A04P01756	A04P11418	5.1077	2.426	-0.0531	-0.0644	-0.0292
205	6	LB	2	30	A02P03360	A02P07438	4	50	A04P13832	RM471	6.1553	6.2886	-0.0371	-0.0002	-0.1127

S. No	ID	Trait	Chr 1	Position 1	Left Marker 1	Right Marker 1	Chr 2	Position 2	Left Marker 2	Right Marker 2	LOD	PVE	Add1	Add2	Add by Add
206	6	LB	4	45	A04P13832	RM471	7	30	RM20818	RM481	6.0358	6.4545	0.0345	-0.0438	0.0216
207	6	LB	8	70	A08P23255	RM22554	9	65	RM13021	A09P12377	5.8726	9.682	-0.0835	0.1583	0.0354
208	6	LB	7	140	RM21652	RM1132	9	75	RM13021	A09P12377	5.175	9.8079	0.0459	0.0443	0.0488
209	6	LB	9	80	RM13021	A09P12377	9	145	A09P09438	A09P18034	5.0436	10.1857	0.1073	-0.119	-0.0248
210	6	LB	6	35	A06P08188	RM3408	9	155	A09P09438	A09P18034	6.6439	5.3637	-0.0652	0.0939	-0.0842
211	6	LB	2	5	A02P01132	A02P03360	10	55	RM25280	RM6737	5.1244	6.1833	-0.051	-0.08	-0.1047
212	6	LB	4	150	RM3367	RM17296	10	175	RM25794	A10P22154	5.8148	2.5939	-0.0116	-0.0036	-0.0334
213	6	LB	10	180	RM25794	A10P22154	11	225	RM13347	RM206	5.2359	2.7795	0.0067	0.0014	-0.0535
214	6	LB	4	85	RM471	RM17201	12	25	A12P22450	A12P05053	5.7137	8.0606	-0.1373	-0.0015	-0.0551
215	8	PTPP	1	235	RM11307	RM10890	1	450	RM297	RM11935	6.6761	2.5583	2.746	-1.3276	0.9242
216	8	PTPP	1	155	RM562	RM11307	2	105	RM6165	RM341	7.1332	2.7706	-2.6934	3.9755	-1.1376
217	8	PTPP	2	110	RM6165	RM341	2	130	RM341	RM13601	7.2532	1.7457	-1.253	1.3097	-2.4203
218	8	PTPP	1	145	RM562	RM11307	3	150	A03P27107	RM85	9.253	2.8249	2.2822	2.7832	2.2156
219	8	PTPP	2	155	RM341	RM13601	3	155	A03P27107	RM85	6.4324	2.049	-1.1559	-0.4142	4.818
220	8	PTPP	3	150	A03P27107	RM85	3	160	A03P27107	RM85	8.4001	2.7748	-1.037	2.5425	-5.1678
221	8	PTPP	3	195	RM85	RM16097	4	0	A04P01363	A04P01756	6.3651	1.7337	0.0605	0.0965	-5.0561
222	8	PTPP	2	210	RM263	RM5470	4	175	RM241	RM127	5.0764	1.868	2.9144	-1.9985	-1.0611
223	8	PTPP	3	190	RM85	RM16097	5	30	A05P00597	A05P05283	7.1879	2.2365	1.8462	-3.4412	-0.9035
224	8	PTPP	1	145	RM562	RM11307	5	110	A05P22287	A05P26105	5.6624	2.1101	-0.327	-2.3525	0.5174
225	8	PTPP	3	205	RM85	RM16097	6	20	A06P22133	A06P08188	5.1021	1.7321	-0.6999	1.2369	3.4343
226	8	PTPP	1	165	RM562	RM11307	7	10	RM20818	RM481	6.5245	2.2064	-0.1363	2.2538	0.8048
227	8	PTPP	3	150	A03P27107	RM85	7	20	RM20818	RM481	6.2682	2.596	-4.2851	1.5175	-2.018
228	8	PTPP	4	95	RM471	RM17201	8	20	RM23556	A08P25335	5.9772	1.7157	-1.5716	1.81	-2.1914
229	8	PTPP	1	170	RM562	RM11307	8	45	A08P25335	A08P23255	8.9138	2.3916	1.6315	2.1454	1.0887
230	8	PTPP	3	195	RM85	RM16097	8	70	A08P23255	RM22554	7.3597	2.6602	3.0658	1.5601	1.9531
231	8	PTPP	5	110	A05P22287	A05P26105	9	20	RM13021	A09P12377	6.0166	1.8467	0.8109	1.236	0.0938
232	8	PTPP	1	160	RM562	RM11307	9	40	RM13021	A09P12377	8.5228	2.988	-1.0044	1.9663	-2.6611
233	8	PTPP	3	150	A03P27107	RM85	9	75	RM13021	A09P12377	6.864	2.6044	-2.4651	-1.8911	3.9897
234	8	PTPP	9	80	RM13021	A09P12377	9	110	A09P12377	RM23914	5.1703	1.9456	3.7302	-3.1209	-2.1842
235	8	PTPP	7	65	RM481	RM21097	10	0	RM216	A10P05672	5.0934	0.798	0.2686	-0.2769	1.9532

S. No	ID	Trait	Chr 1	Position 1	Left Marker 1	Right Marker 1	Chr 2	Position 2	Left Marker 2	Right Marker 2	LOD	PVE	Add1	Add2	Add by Add
236	8	PTPP	1	320	RM10890	RM128	10	50	RM25280	RM6737	6.7379	2.5042	-0.0596	-0.2887	-5.0509
237	8	PTPP	2	165	RM341	RM13601	10	55	RM25280	RM6737	6.1718	1.2001	-0.7294	-2.838	1.5358
238	8	PTPP	3	145	A03P27107	RM85	10	60	RM25280	RM6737	8.3493	2.4704	0.1798	-0.4334	-3.8256
239	8	PTPP	10	160	RM6100	RM25794	10	170	RM25794	A10P22154	6.548	1.7037	-0.9571	0.3983	-3.957
240	8	PTPP	11	10	RM287	A11P05095	11	15	RM287	A11P05095	5.3922	1.8103	-0.3853	-0.7468	-0.3008
241	8	PTPP	4	80	RM471	RM17201	11	45	A11P05095	RM12568	5.5258	1.6085	0.947	-0.8055	3.3726
242	8	PTPP	1	155	RM562	RM11307	11	55	A11P05095	RM12568	7.1732	2.5021	-0.7087	1.1496	-3.5985
243	8	PTPP	7	60	RM481	RM21097	11	55	A11P05095	RM12568	5.8287	1.7993	-2.4531	2.3516	-1.5446
244	8	PTPP	9	70	RM13021	A09P12377	11	55	A11P05095	RM12568	8.4574	2.8472	-4.0415	2.5067	-0.1903
245	8	PTPP	5	115	A05P22287	A05P26105	11	75	A11P05095	RM12568	5.339	2.1373	0.7809	0.987	0.5176
246	8	PTPP	8	50	A08P25335	A08P23255	11	75	A11P05095	RM12568	5.3093	2.0365	2.564	-1.7913	-0.4607
247	8	PTPP	2	110	RM6165	RM341	11	170	RM13347	RM206	7.9893	1.8854	0.7102	-0.4995	-2.9442
248	8	PTPP	3	155	A03P27107	RM85	11	200	RM13347	RM206	9.4527	2.738	3.0488	1.8385	1.836
249	8	PTPP	10	60	RM25280	RM6737	12	10	A12P22450	A12P05053	5.9616	2.471	0.9856	2.7465	2.9236
250	8	PTPP	3	150	A03P27107	RM85	12	25	A12P22450	A12P05053	7.6548	2.6338	-2.8027	3.6212	1.472
251	8	PTPP	2	210	RM263	RM5470	12	65	A12P05053	RM27564	5.7714	1.7189	-4.2325	1.2143	-0.5155
252	8	PTPP	5	115	A05P22287	A05P26105	12	130	A12P02180	RM235	6.6535	2.1577	0.3142	0.9945	-0.2961
253	8	PTPP	9	70	RM13021	A09P12377	12	130	A12P02180	RM235	6.3339	2.8838	2.2626	1.5877	1.5989
254	8	PTPP	11	70	A11P05095	RM12568	12	145	A12P02180	RM235	5.6567	2.6735	1.978	1.887	4.7649
255	8	PTPP	1	155	RM562	RM11307	12	150	A12P02180	RM235	8.9222	2.9217	-1.0862	2.1282	-2.3693
256	8	PTPP	12	130	A12P02180	RM235	12	155	A12P02180	RM235	5.9666	2.4547	0.4484	0.7348	0.2264
257	12	GYPP	1	190	RM562	RM11307	1	375	RM128	RM1297	10.6644	1.3436	-8.7728	-6.5619	8.4996
258	12	GYPP	1	235	RM11307	RM10890	2	105	RM6165	RM341	9.683	1.4243	-6.7879	4.4409	-10.8589
259	12	GYPP	2	140	RM341	RM13601	2	150	RM341	RM13601	9.6512	1.1875	5.5201	-4.5068	-10.4733
260	12	GYPP	2	205	RM263	RM5470	3	25	RM22	A03P09039	8.2663	1.1795	8.3954	-8.8851	-3.2547
261	12	GYPP	1	205	RM11307	RM10890	3	200	RM85	RM16097	10.3581	1.6998	-7.9934	9.7863	-6.0132
262	12	GYPP	3	150	A03P27107	RM85	3	200	RM85	RM16097	10.1566	1.6688	-10.7752	7.681	-2.8767
263	12	GYPP	3	200	RM85	RM16097	4	45	A04P13832	RM471	8.9997	1.2191	5.4923	-5.8471	-9.9974
264	12	GYPP	4	40	A04P13832	RM471	4	60	A04P13832	RM471	8.2883	0.5614	9.2462	-10.3128	-9.737
265	12	GYPP	2	215	RM263	RM5470	4	175	RM241	RM127	7.7839	1.2578	12.0972	-7.3048	-4.4926

S. No	ID	Trait	Chr 1	Position 1	Left Marker 1	Right Marker 1	Chr 2	Position 2	Left Marker 2	Right Marker 2	LOD	PVE	Add1	Add2	Add by Add
266	12	GYPP	1	260	RM11307	RM10890	4	180	RM241	RM127	7.73	1.3752	-12.6097	-6.3421	3.6775
267	12	GYPP	4	180	RM241	RM127	5	65	A05P10927	RM164	6.5279	0.9074	-9.4338	-8.0501	8.9575
268	12	GYPP	1	210	RM11307	RM10890	5	115	A05P22287	A05P26105	8.8815	1.1247	-7.6626	8.9634	-6.8969
269	12	GYPP	3	205	RM85	RM16097	5	115	A05P22287	A05P26105	7.5608	1.2026	5.4605	5.9761	10.438
270	12	GYPP	2	40	A02P03360	A02P07438	5	135	A05P26105	A05P25260	7.6194	0.9423	-8.9772	7.2698	-5.5537
271	12	GYPP	5	120	A05P22287	A05P26105	5	135	A05P26105	A05P25260	9.5727	1.0415	0.2409	0.5383	1.9636
272	12	GYPP	3	200	RM85	RM16097	6	10	A06P22133	A06P08188	8.5151	1.447	4.0628	9.9089	6.0526
273	12	GYPP	1	215	RM11307	RM10890	6	15	A06P22133	A06P08188	9.5841	1.2429	-7.8889	6.4515	-8.8882
274	12	GYPP	2	30	A02P03360	A02P07438	6	15	A06P22133	A06P08188	7.5715	0.8686	-6.6671	7.6565	-6.1223
275	12	GYPP	4	175	RM241	RM127	6	30	A06P08188	RM3408	5.5247	0.91	-6.5618	10.0256	-6.3513
276	12	GYPP	6	10	A06P22133	A06P08188	6	45	RM3408	RM586	9.3409	1.0222	9.055	-9.2275	-5.3436
277	12	GYPP	5	135	A05P26105	A05P25260	6	60	RM3408	RM586	5.0641	0.9228	9.1073	-7.6851	-6.643
278	12	GYPP	3	25	RM22	A03P09039	7	0	RM20818	RM481	7.5078	0.8657	-0.1063	-1.4792	-0.2643
279	12	GYPP	4	135	RM3367	RM17296	7	60	RM481	RM21097	9.2994	0.9921	-6.3118	7.3401	-9.6957
280	12	GYPP	1	205	RM11307	RM10890	7	85	RM21097	RM21577	9.5825	1.1116	-7.4615	8.0986	-6.7508
281	12	GYPP	2	155	RM341	RM13601	7	85	RM21097	RM21577	7.7781	1.0127	-5.6908	6.8259	-5.1984
282	12	GYPP	6	10	A06P22133	A06P08188	7	90	RM21097	RM21577	6.5078	1.1309	5.1213	7.0656	7.1037
283	12	GYPP	7	85	RM21097	RM21577	7	120	RM21577	RM21652	8.1965	1.0258	9.2108	-7.3562	-6.6312
284	12	GYPP	1	185	RM562	RM11307	8	10	RM23556	A08P25335	9.4102	1.1748	-6.4675	-6.8742	8.6366
285	12	GYPP	8	35	A08P25335	A08P23255	8	45	A08P25335	A08P23255	8.3336	0.9149	-10.5216	9.7006	-9.3642
286	12	GYPP	6	65	RM3408	RM586	8	70	A08P23255	RM22554	9.4703	1.1726	-6.6076	4.6068	-10.1952
287	12	GYPP	3	140	A03P27107	RM85	8	75	A08P23255	RM22554	8.076	1.2253	-5.4127	6.5285	-10.951
288	12	GYPP	4	175	RM241	RM127	8	75	A08P23255	RM22554	6.9651	1.2379	5.7681	4.2792	9.5151
289	12	GYPP	2	200	RM263	RM5470	8	80	A08P23255	RM22554	6.8751	1.5096	5.6805	-12.5407	-4.8656
290	12	GYPP	4	55	A04P13832	RM471	9	10	RM13021	A09P12377	8.6692	0.9562	-7.4201	9.5838	-6.8209
291	12	GYPP	7	70	RM481	RM21097	9	35	RM13021	A09P12377	7.8096	1.6221	7.5352	9.0161	3.53
292	12	GYPP	3	150	A03P27107	RM85	9	45	RM13021	A09P12377	10.633	2.0998	3.0405	6.801	12.1014
293	12	GYPP	6	35	A06P08188	RM3408	9	60	RM13021	A09P12377	9.1025	1.8795	6.7898	11.6566	7.4167
294	12	GYPP	8	70	A08P23255	RM22554	9	65	RM13021	A09P12377	7.6671	1.6389	5.5708	-8.3569	-5.1505
295	12	GYPP	2	200	RM263	RM5470	9	75	RM13021	A09P12377	11.4131	2.1256	2.8766	6.8489	11.744

S. No	ID	Trait	Chr 1	Position 1	Left Marker 1	Right Marker 1	Chr 2	Position 2	Left Marker 2	Right Marker 2	LOD	PVE	Add1	Add2	Add by Add
296	12	GYPP	5	135	A05P26105	A05P25260	9	75	RM13021	A09P12377	8.7224	1.3701	7.0816	5.2313	7.87
297	12	GYPP	9	25	RM13021	A09P12377	9	75	RM13021	A09P12377	13.0979	2.1899	5.2325	9.1116	9.2921
298	12	GYPP	1	205	RM11307	RM10890	9	80	RM13021	A09P12377	12.8382	1.8234	-5.0736	5.453	-9.0628
299	12	GYPP	1	240	RM11307	RM10890	10	45	RM25280	RM6737	8.8502	1.1643	-8.3091	-6.4649	5.2197
300	12	GYPP	3	185	RM85	RM16097	10	45	RM25280	RM6737	8.5185	1.2815	5.3034	-4.3849	-8.877
301	12	GYPP	4	50	A04P13832	RM471	10	50	RM25280	RM6737	9.042	1.1322	-5.8351	-7.0664	8.9051
302	12	GYPP	5	135	A05P26105	A05P25260	10	50	RM25280	RM6737	6.6636	0.8595	7.0408	-7.9866	-6.4025
303	12	GYPP	6	15	A06P22133	A06P08188	10	50	RM25280	RM6737	8.5945	1.0526	5.317	-7.5011	-8.3324
304	12	GYPP	8	50	A08P25335	A08P23255	10	65	RM25280	RM6737	6.7321	1.1175	6.9009	-5.7523	-5.2876
305	12	GYPP	9	75	RM13021	A09P12377	10	65	RM25280	RM6737	12.0937	2.2032	9.6637	-4.6002	-9.3121
306	12	GYPP	2	195	RM263	RM5470	10	70	RM25280	RM6737	8.6579	1.1929	8.1894	-7.625	-3.7789
307	12	GYPP	7	20	RM20818	RM481	10	165	RM6100	RM25794	8.8512	1.0564	3.1516	-3.7964	-6.8415
308	12	GYPP	10	160	RM6100	RM25794	10	170	RM25794	A10P22154	8.952	1.2576	7.5215	-7.2965	-13.3276
309	12	GYPP	1	200	RM11307	RM10890	11	15	RM287	A11P05095	11.0027	1.1999	-6.935	8.632	-5.4249
310	12	GYPP	3	195	RM85	RM16097	11	15	RM287	A11P05095	9.6901	1.5077	5.3288	9.95	4.1007
311	12	GYPP	4	170	RM241	RM127	11	15	RM287	A11P05095	7.5394	1.1643	4.1847	11.3402	4.2899
312	12	GYPP	6	60	RM3408	RM586	11	15	RM287	A11P05095	8.0954	1.176	-8.4203	5.488	-9.3874
313	12	GYPP	8	75	A08P23255	RM22554	11	15	RM287	A11P05095	7.2437	1.1203	8.2361	6.6975	9.6009
314	12	GYPP	5	30	A05P00597	A05P05283	11	20	RM287	A11P05095	7.1295	1.0919	-6.2317	7.4733	-8.404
315	12	GYPP	7	20	RM20818	RM481	11	20	RM287	A11P05095	7.6842	1.1271	5.3712	8.8428	5.685
316	12	GYPP	9	145	A09P09438	A09P18034	11	20	RM287	A11P05095	9.5703	1.4623	4.3624	8.8338	6.1248
317	12	GYPP	10	155	RM6100	RM25794	11	20	RM287	A11P05095	11.786	1.2158	-5.4967	3.7931	-6.2388
318	12	GYPP	11	25	RM287	A11P05095	11	40	A11P05095	RM12568	10.8776	1.3637	8.6417	-10.517	-10.411
319	12	GYPP	2	100	RM6165	RM341	11	155	RM12568	RM13347	10.8844	1.2454	7.4173	-7.4026	-8.4788
320	12	GYPP	10	160	RM6100	RM25794	12	15	A12P22450	A12P05053	7.3221	0.9983	-5.9847	6.6042	-4.7027
321	12	GYPP	6	50	RM3408	RM586	12	80	RM27564	A12P02180	7.938	1.0633	-6.8628	6.7845	-6.5932
322	12	GYPP	12	50	A12P05053	RM27564	12	80	RM27564	A12P02180	10.5244	1.0577	-9.064	8.0906	-5.5423
323	12	GYPP	9	75	RM13021	A09P12377	12	85	RM27564	A12P02180	8.9493	1.3864	10.0935	8.6189	6.7933
324	12	GYPP	4	50	A04P13832	RM471	12	90	RM27564	A12P02180	7.9406	1.0069	-5.9429	7.5062	-6.1773
325	12	GYPP	8	70	A08P23255	RM22554	12	130	A12P02180	RM235	7.9333	1.749	4.9368	13.1868	5.0074

S. No	ID	Trait	Chr 1	Position 1	Left Marker 1	Right Marker 1	Chr 2	Position 2	Left Marker 2	Right Marker 2	LOD	PVE	Add1	Add2	Add by Add
326	12	GYPP	3	80	RM232	A03P23446	12	135	A12P02180	RM235	10.2847	2.1981	-4.994	-4.4094	13.555
327	12	GYPP	7	15	RM20818	RM481	12	135	A12P02180	RM235	7.1034	1.6887	3.3425	11.2567	6.5899
328	12	GYPP	1	185	RM562	RM11307	12	140	A12P02180	RM235	11.4046	1.7557	-8.7824	8.635	-7.3881
329	12	GYPP	2	200	RM263	RM5470	12	140	A12P02180	RM235	8.8782	1.9559	2.3029	11.4316	6.9771
330	12	GYPP	11	200	RM13347	RM206	12	140	A12P02180	RM235	8.552	1.9822	-6.1083	-4.3018	14.063
331	12	GYPP	5	95	RM164	A05P22287	12	155	A12P02180	RM235	6.7678	1.0419	-6.6542	9.5436	-6.9168
